# Specific *Cryptosporidium* antigens associate with reinfection immunity and protection from cryptosporidiosis

**DOI:** 10.1172/JCI166814

**Published:** 2023-08-15

**Authors:** Carol A. Gilchrist, Joseph J. Campo, Jozelyn V. Pablo, Jennie Z. Ma, Andy Teng, Amit Oberai, Adam D. Shandling, Masud Alam, Mamun Kabir, A.S.G. Faruque, Rashidul Haque, William A. Petri

**Affiliations:** 1 Department of Medicine, University of Virginia, Charlottesville, Virginia, USA.; 2 Antigen Discovery Inc, Irvine, California, USA.; 3 Public Health Science, University of Virginia, Charlottesville, Virginia, USA.; 4 International Centre for Diarrhoeal Diseases Research, Dhaka, Bangladesh.; 5 Microbiology, Immunology and Cancer Biology, and; 6 Pathology, University of Virginia, Charlottesville, Virginia, USA.

**Keywords:** Immunology, Infectious disease, Adaptive immunity, Parasitology

## Abstract

There is no vaccine to protect from cryptosporidiosis, a leading cause of diarrhea in infants in low- and middle-income countries. Here, we comprehensively identified parasite antigens associated with protection from reinfection. A *Cryptosporidium* protein microarray was constructed by in vitro transcription and translation of 1,761 *C*. *parvum*, *C*. *hominis*, or *C*. *meleagridis* antigens, including proteins with a signal peptide and/or a transmembrane domain. Plasma IgG and/or IgA from Bangladeshi children longitudinally followed for cryptosporidiosis from birth to 3 years of age allowed for identification of 233 seroreactive proteins. Seven of these were associated with protection from reinfection. These included Cp23, Cp17, Gp900, and 4 additional antigens — CpSMP1, CpMuc8, CpCorA and CpCCDC1. Infection in the first year of life, however, often resulted in no detectable antigen-specific antibody response, and antibody responses, when detected, were specific to the infecting parasite genotype and decayed in the months after infection. In conclusion, humoral immune responses against specific parasite antigens were associated with acquired immunity. While antibody decay over time and parasite genotype-specificity may limit natural immunity, this work serves as a foundation for antigen selection for vaccine design.

## Introduction

*Cryptosporidium* parasites are a common cause of diarrhea in infants and children in low- and middle-income countries, globally are the most common cause of outbreaks of waterborne disease, and, in immunocompromised individuals, can cause protracted infections (1). Evidence that a vaccine is an achievable goal includes that the incidence of infection declines with age (2–4) and that antibodies against antigens on the surface of the infectious oocyst (5) and invasive sporozoite parasite life stages are associated with partial immunity to reinfection (6, 7). While the associated protective immune response involves both cellular and humoral immunity, identification of antibody responses against *Cryptosporidium* antigens associated with protection from reinfection may provide a foundation for the development of an effective anti-*Cryptosporidium* vaccine.

We previously conducted a natural history study of cryptosporidiosis in infants longitudinally followed for infection from birth to 3 years of age (National Clinical Trial Identifier: NCT02764918) (8–11). The infants resided in a low income community of Dhaka, Bangladesh. Fecal DNA was extracted from diarrheal and monthly surveillance stool samples and symptomatic and asymptomatic cases of cryptosporidiosis were identified using a pan-*Cryptosporidium* qPCR assay. By the end of the first year, 27.5% (*n* = 109) of infants had been infected at least once. The immunity arising from this infection was, however, incomplete, with most of these children reinfected by 3 years of age (Supplemental Figure 1; supplemental material available online with this article; https://doi.org/10.1172/JCI166814DS1) (9). These repeat infections had a lower burden of parasites and were more likely to be subclinical in nature.

Here, we present the results from probing a *Cryptosporidium* protein microarray with antisera from 1-year-old infants. The association of antibody responses against specific proteins with immunity to reinfection at 2 and 3 years of age was then tested.

## Results

### Identification of immunoreactive proteins.

The *Cryptosporidium* species protein microarray created for this study comprised a total of 1,761 antigens representing 1,250 unique genes from *C*. *parvum* (*n* = 980), *C*. *hominis* (*n* =263), and *C*. *meleagridis* (*n* =7). *C*. *parvum* sequences were used as the backbone of the protein microarray due to the ready availability of *C*. *parvum* DNA and its superior assembly and annotation (12, 13) (Supplemental Figure 2). The selected *C*. *parvum* proteins included those that had been previously identified as potential vaccine candidates (Supplemental Table 1) (6, 14–34). Open reading frames (ORFs) over 3,000 base pairs were cloned as overlapping segments to optimize in vitro translation. The array also included 15 genetically variant regions of the *gp60* gene common in this Bangladeshi population (35). Proteins with conserved sequences in the different *Cryptosporidium* species that account for the majority of cryptosporidiosis in humans (*C*. *hominis*, *C*. *parvum* and *C*. *meleagridis*) and that were annotated as having a signal peptide — and, thus, potentially a membrane protein and accessible to human antibodies — were prioritized for inclusion (Supplemental Figure 3).

The array was incubated with a 1:100 dilution of plasma collected at 1 year of age from 500 children in the cohort and developed with anti-human IgG (DyLight650, Bethyl Laboratories) and Cy3 AffiniPure F(ab′)_2_ or anti-human IgA. The distribution of normalized fluorescence signal intensity (SI) values of each antigen was analyzed using a mixture modeling technique Supplemental Figure 4A) to identify the antigen specific background component of the spot signal and hence the appropriate intensity, or cut-off value, that was defined as seropositive (Supplemental Figure 4B).

**Figure 1:**
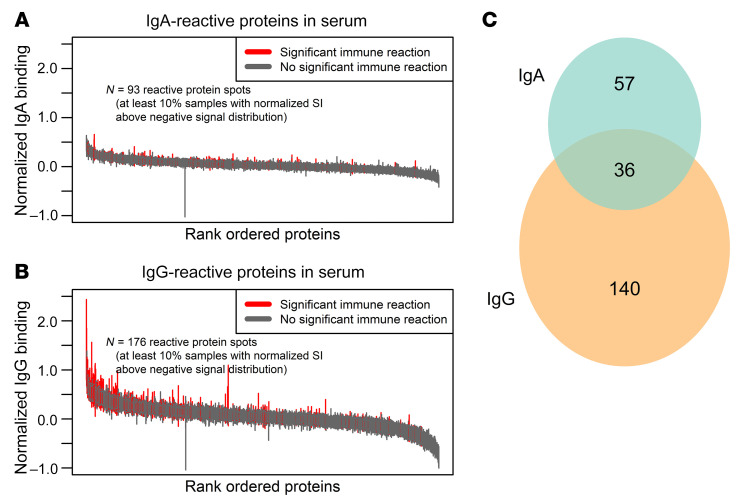
Humoral immunity to *Cryptosporidium* antigens was isotype specific. Immune responses are shown for (**A**) IgA and (**B**) IgG antibodies. The Y-axis is signal intensity after normalization and the X-axis shows *Cryptosporidium* antigens ranked by median signal intensity. Bars represent the interquartile range of each antibody response and are shown as red if antibodies were present in ≥ 10% of infants (seroprevalent). (**C**) The Venn diagram shows seroprevalent *Cryptosporidium* antigens with IgA- (green) and IgG-specific (orange) and overlapping immune responses.

**Figure 2:**
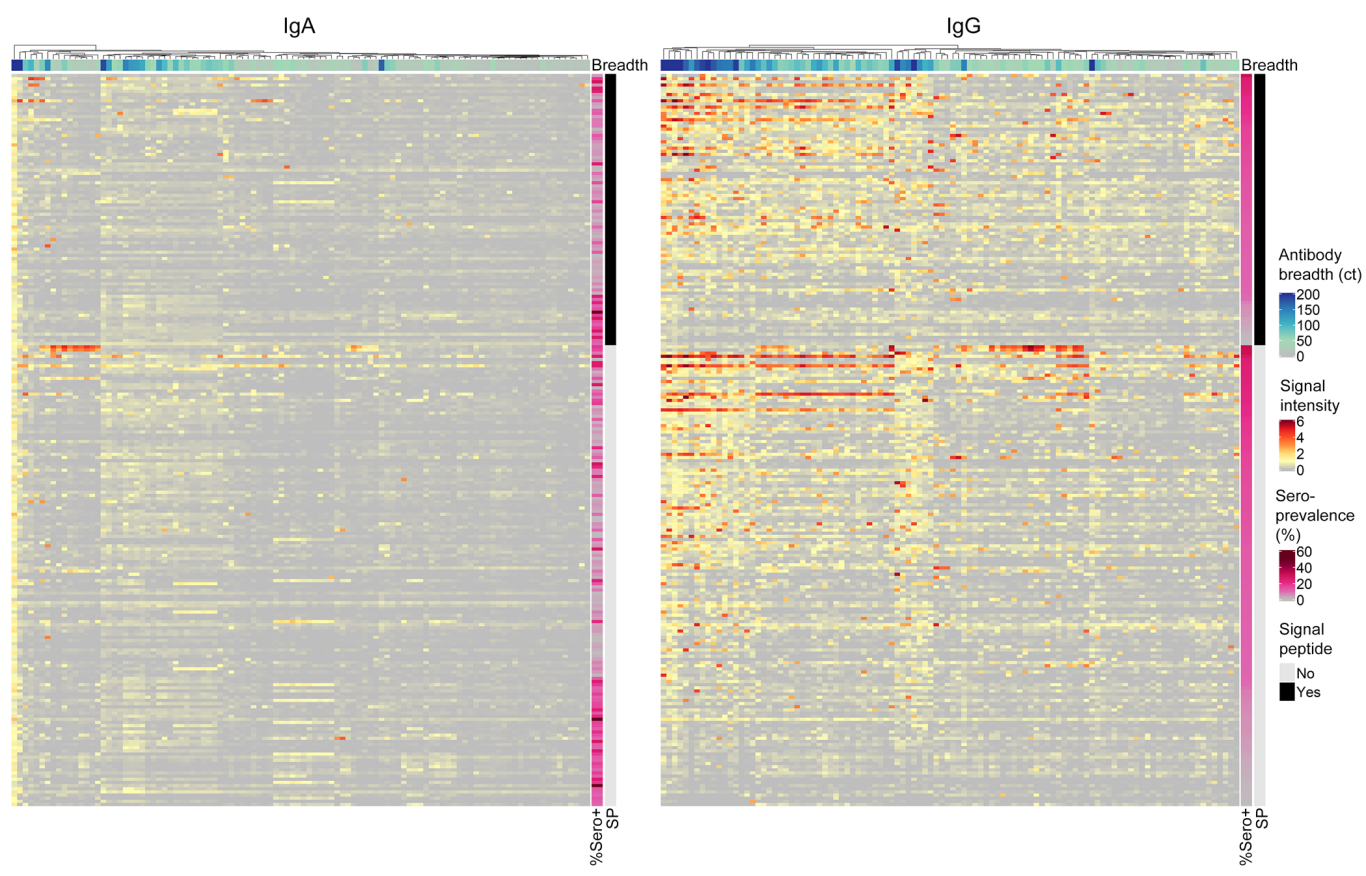
*Cryptosporidium* antigens recognized by IgA and IgG antibodies. The proteomic microarray was used to measure the parasite-specific antibody response in the infants enrolled in our study cohort at 1 year in age. Previously infected children (columns) and the *Cryptosporidium* antigens (rows) that stimulated a strong IgG and/or IgA antibody response (present in > 10% of the children; *n* =232 antigens) are shown. The spot signals were normalized by first determining the specific background component by use of mixture models and setting this value to 0. Bar at the top of each heat map indicates the total number of *Cryptosporidium* antigens each child responds to (Antibody Breadth). The side bars indicate: (a) the seroprevalence of each antigen (% Sero+) and (b) presence of a membrane-targeting signal peptide (SP).

Antigens were classified as seroreactive in this population if at least 10% of the children had either IgA (Figure 1A) or IgG (Figure 1B) antibodies against the antigen. Using this criterion, 36 antigens were seroreactive to both IgG and IgA (Supplemental Figure 5), 57 antigens by IgA alone, and 140 antigens by IgG alone (Figure 1C). Antigens recognized varied greatly between children, with each antigen recognized by only a subset of the responding infants (Figure 2).

We found that orthologues encoded by *C*. *parvum* and *C*. *hominis* generated similar signals; for example the *C*. *parvum* Cp23 (cgd4_3620) and the *C*. *hominis* Cp23 orthologue (Chro.40414) signals were correlated (Pearson *r* value 0.844 *P* = 2.11 × 10^–119^). In total, there were 124 IgG-reactive proteins and 70 IgA-reactive *C*. *parvum* proteins. Antigens recognized were from multiple developmental stages of the *Cryptosporidium* parasite (36–38) (Supplemental Figure 2).

### Humoral immune response diminished with time from infection.

**Figure 3:**
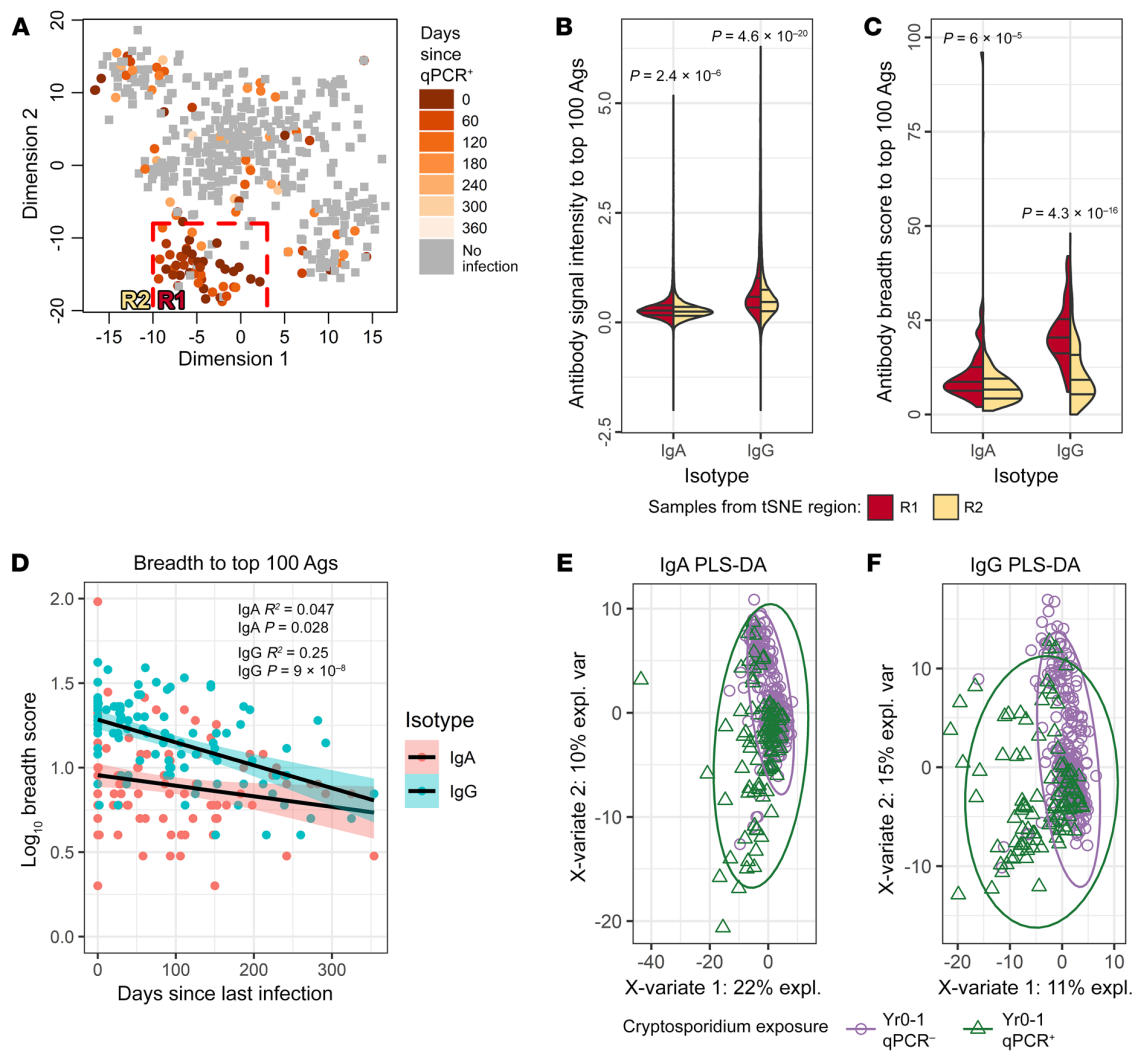
Antibody responses waned with time after a *Cryptosporidium* infection. (**A**) The t-SNE plot identified a subset of children with a similar antibody profile. Each point corresponds to the immune profile of a child. Gray squares indicate children where no previous *Cryptosporidium* infections were identified by qPCR in clinical or surveillance stool samples (“qPCR^–^”), and orange circles represent children that had previous infections detected by qPCR (“qPCR^+^”), with the intensity of the overlaid color indicating the days since the last *Cryptosporidium* qPCR^+^ stool sample was identified. A group of infants had similar antibody profiles and a high density of recent infections (R1). (**B**) The split violin plot of antibody signals against the 100 most-reactive antigens (Y-axis) for each isotype (X-axis) shows the responses of children within the R1 region of the t-SNE plot compared with the remainder of the samples in R2. The median and quartile values are shown as horizontal lines in each split violin. (**C**) The split violin plot shows the same comparison as (**B**) using the antibody breadth (count of seropositive responses) among the 100 most-reactive antigens. *P* values above each split violin were calculated using linear mixed effects regression (LMER) and Wilcoxon’s rank sum tests for (**B** and **C**), respectively. (**D**) Antibody breadth among the 100 most-reactive antigens for each isotype is shown on the Y-axis after log_10_ transformation with the interval (days) between the last *Cryptosporidium* qPCR^+^ diagnostic assay and the time of antibody measurement shown on the X-axis. Linear regression *P* values and R^2^ values are shown for IgG and IgA, as well as a line and confidence intervals (colored bands; pink for IgA and green for IgG) fit to each. (**E** and **F**) PLS-DA is shown for IgA and IgG responses respectively. Each point corresponds to the immune profile of a child. The purple circles indicate the antibody response obtained from plasma that was collected from children where none of the stool samples (diarrheal or surveillance) collected during the first year of life, prior to the plasma sampling time point, were ever qPCR positive for *Cryptosporidium* parasites (“Yr0-1 qPCR^–^”). Green triangles indicate that the child had a verified *Cryptosporidium* subclinical or symptomatic infection (“Yr-0-1 qPCR^+^”). The percentage of the variation in the child’s antibody profile accounted for by each axis is indicated.

The anti-*Cryptosporidium* IgA and IgG antibody profiles were analyzed using *t*-distributed stochastic neighbor embedding (t-SNE) as an unsupervised data reduction method for visualization of the trends in the antibody profile, based on the number of days since the first *Cryptosporidium*-positive diagnostic qPCR and whether the child had a documented prior *Cryptosporidium* infection (Figure 3A). The antibody profile of children with more recent infections mapped to a distinct region within the t-SNE plot (Region 1, “R1”), while children with earlier infections did not cluster separately from children without a prior infection (Figure 3A). By analyzing the 100 most responsive antigens, the important factors that influenced the antibody profile of children in R1 of the t-SNE plot were the strength of the immune response (Figure 3B) and its breadth, or the number of parasite antigens recognized by IgA and IgG (Figure 3C) (39). Diminishing antibody responses over time were confirmed by a linear regression analysis where, most notably, the breadth of both the IgA and IgG anti-*Cryptosporidium* immune responses significantly decreased over time (Figure 3D).

### Impact of prior infection on antibody response.

While the effect of time since *Cryptosporidium* infection on antibody levels was significant, it did not completely explain the failure to generate an anti-*Cryptosporidium* immune response in all cases. To focus on the impact of prior exposure to the *Cryptosporidium* parasite, the data were analyzed using partial least squares discriminant analysis (PLS-DA) (Figure 3, E and F). This analysis demonstrated that a subset of children with prior infection differed from the population of children with no prior infection, but that a substantial proportion of children with an earlier infection had antibody profiles similar to children without a documented prior infection. We concluded that some infections in this cohort were missed, despite the active surveillance system in place.

To explore whether malnutrition or inflammation impacted the humoral immune response, we examined whether the immune profile correlated with growth failure — a measure of chronic malnutrition measured by child height-for-age Z scores (HAZ) — or biomarkers of systemic and local inflammation (sCD14, IL-1Beta, CRP), or immunoregulatory cytokines (IL-4), but no correlations were observed (Supplemental Figure 6).

### Protection from reinfection was not associated with the breadth of the antibody response, i.e., the number of antigens recognized by a given child.

**Figure 4:**
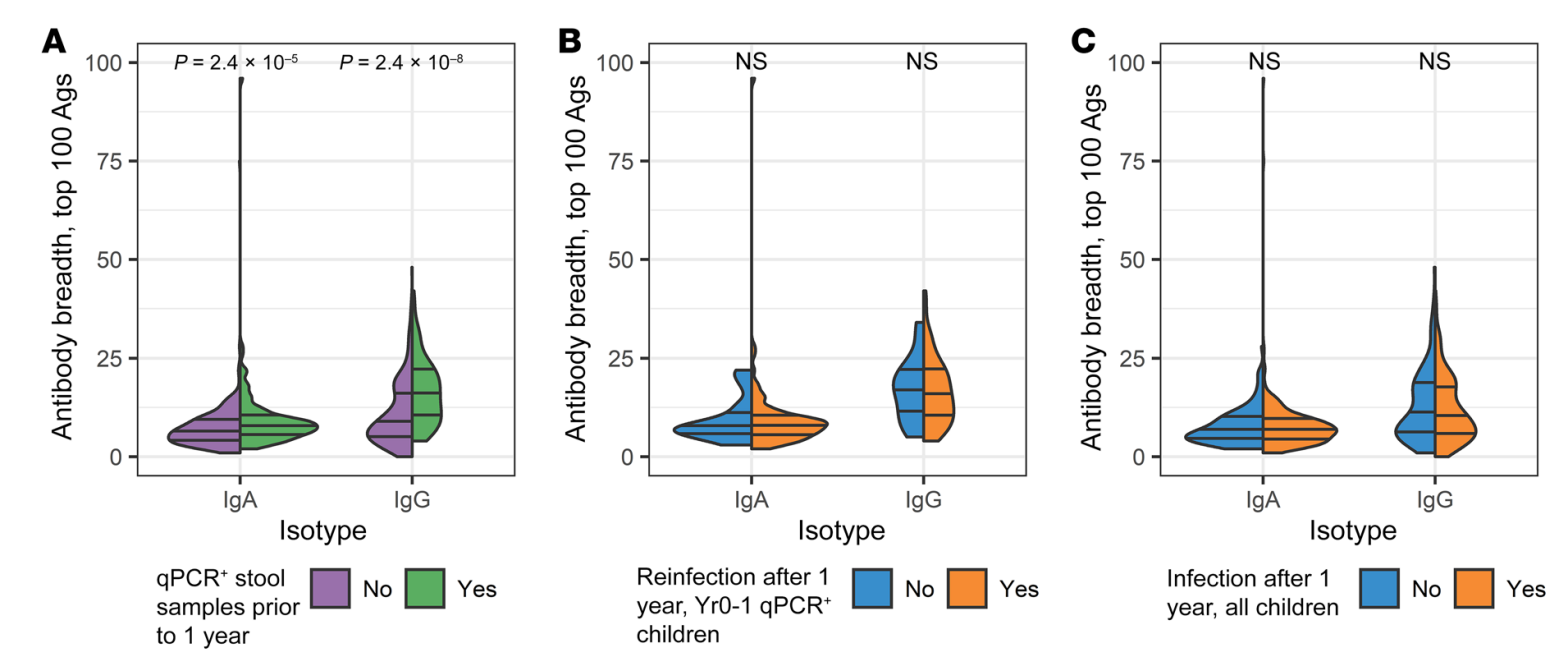
The breadth of the anti-*Cryptosporidium* immune response was not correlated with protection from infection. (**A**) Split violin plot of antibody breadth in plasma among the 100 most-reactive antigens (Y-axis) for each isotype (X-axis) is shown for the comparison between children that had no stool samples (diarrheal or surveillance) qPCR^+^ for *Cryptosporidium* parasites (purple) and children who had a verified *Cryptosporidium* infection (green). (**B**) Data is shown from one year old infants who had prior qPCR-confirmed *Cryptosporidium* infections (“Yr0-1 qPCR^+^”) that were subsequently uninfected (blue) or reinfected (orange) during the next 2 years. (**C**) Data is shown from 1-year-old infants that included both the immunologically naive infants with no prior *Cryptosporidium* infections detected by qPCR in stool samples (diarrheal or surveillance) as well as those with qPCR^+^ stool samples during the first year of life. Medians and quartiles are indicated by horizontal lines in each split violin. Significant *P* values from Wilcoxon’s rank sum tests are shown above violins.

Children for whom a previous infection had been identified had a greater number of parasite antigens recognized by IgA and IgG, or greater “breadth” (Figure 4A). However, no association was found between the breadth of the antibody response and resistance to reinfection using either a data set restricted to the infants with a qPCR-verified *Cryptosporidium* infection prior to year 1 (Figure 4B) or using the data from the entire study cohort (Figure 4C).

### Validation of the array by examining the data obtained from antigens previously associated with a protective immune response.

**Figure 5:**
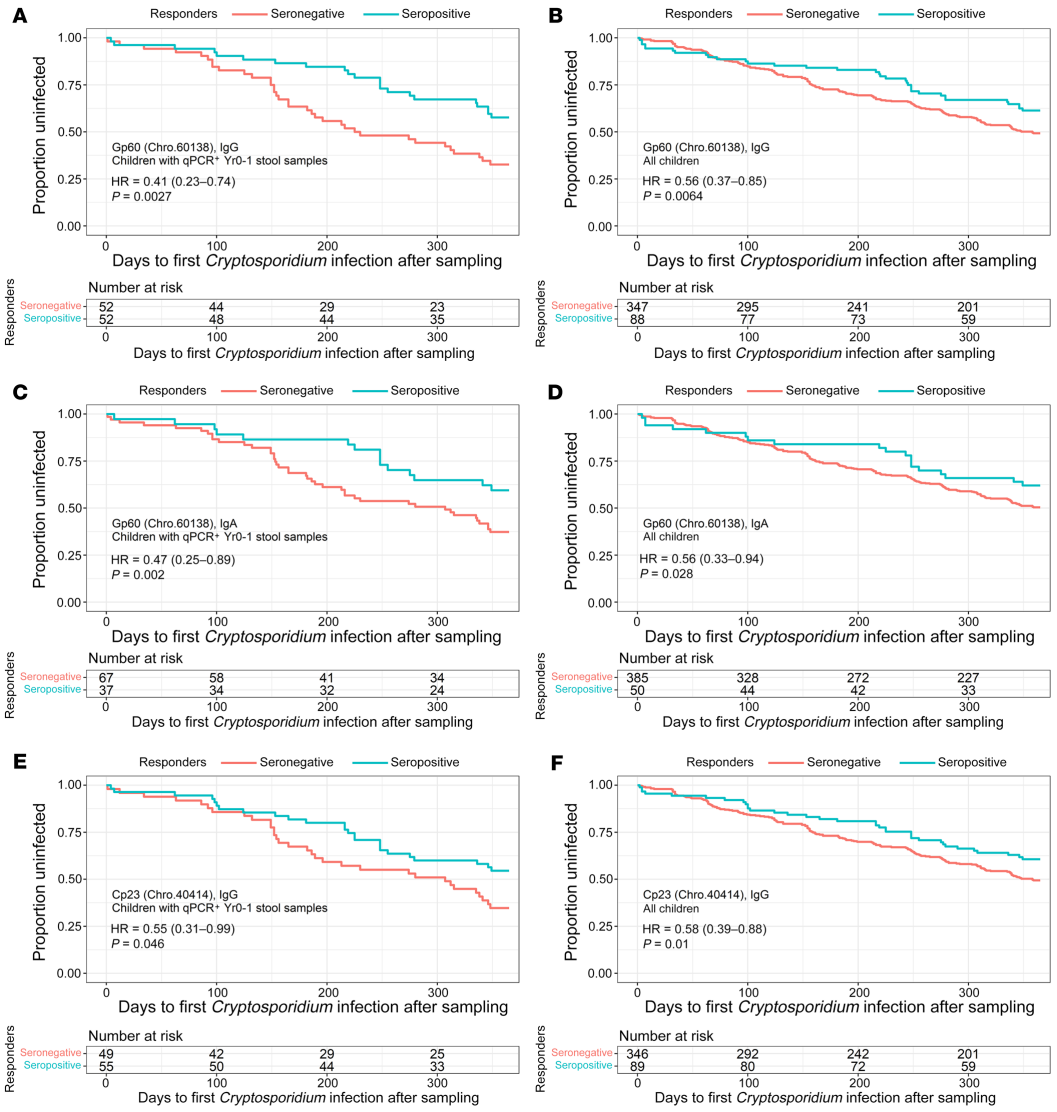
Children with antibodies that targeted the *C*. *hominis* peptides encoded by the *gp60* gene and Cp23 protein were associated with protection from reinfection. In the protein array data, IgA and IgG antibodies against the protein encoded by the *C*. *hominis gp60* gene (Chro.60183) and IgG against Cp23 (Chro.40414) were associated with a delay in *Cryptosporidium* reinfection among children with a qPCR-verified *Cryptosporidium* infection during the first year of life (**A**, **C**, and **E**) or among all children in the study (**B**, **D**, and **F**). The X-axis shows days after the end of year 1 (when the assayed plasma samples were collected). The Y-axis shows the proportion of children who remained uninfected. Red lines represent children seronegative for the antigen, and blue lines represent seropositive children. The Kaplan-Meier curves show the probability of survival free of *Cryptosporidium* species, and the tables below the graphs indicate the number of children in the seropositive or seronegative categories at select time points. (**A** and **B**) IgG against Gp60 (Chro.60183). (**C** and **D**) IgA against Gp60 (Chro.60183). (**E** and **F**) IgG against Cp23 (Chro.40414). Hazard ratios (HR), confidence intervals, and *P* values were calculated using multivariable Cox proportional hazards models.

*C*. *hominis* Cp23 (Chro.40414) and Cp17, a conserved peptide encoded by the variable *C*. *hominis* *gp60* gene (Chro.601380: variant IaA25R3), are both potential vaccine candidates and have been previously shown to be associated with a delay in reinfection in our study population (8, 10, 34). We investigated whether we could also detect an association with protection from reinfection between IgA antibodies recognizing *C*. *hominis* Cp23 (Chro.40414) and the *C*. *hominis* gp60 (Chro.601380) antigens on the *Cryptosporidium* array. As the IgA anti-Cp23 (Chro.40414) signal was low in our array data we were only able to analyze the anti-IgG Cp23 (Chro.40414) data. As expected, a protective association was observed between both anti-IgA and IgG *C*. *hominis* Gp60 (Chro.601380) (Figure 5, A–D) and anti-IgG *C*. *hominis* Cp23 (Chro.40414) (Figure 5, E and F). Since Gp60 and Cp23 were a priori antigen candidates, *P* values were not adjusted for the FDR.

### Impact of the polymorphisms in the gp60 gene on immune reactivity.

**Figure 6:**
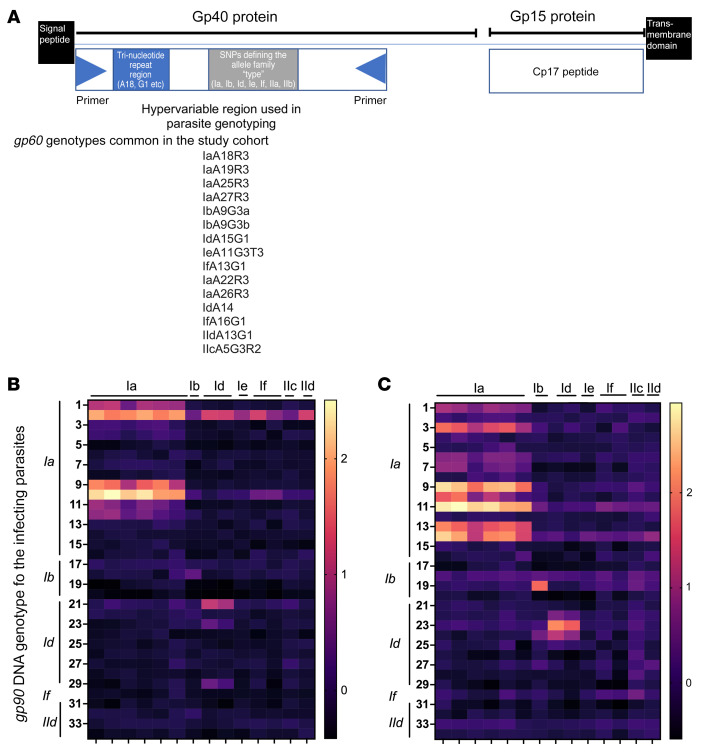
*gp60* Genotype immune response. (**A**) Cartoon illustrating the proteins encoded by the *gp60* gene. (**B** and **C**) Heat maps showing the intensity and breadth of the IgA (**B**) and IgG (**C**) antibody responses to the polymorphic region of the Gp40 protein. The different alleles of the peptide encoded by the *gp60* allele (columns) and the signal obtained when using the plasma with antibodies raised in response to infection of parasite with different *gp60* genotypes (rows). Lines at the top of each heat map indicate the protein type and on the side the genotype of the infecting parasite. Parasite genotypes: rows 1–8: IaA18R3; 9: IaA19R3; 10–16: IaA25R3; 17–20: IbA9G3R2; 21–29: IdA15G1; 30: IfA13G1; 31–34: *C. parvum* IIdA15G1R1. Protein alleles: columns A: IaA27R3, B: IaA26R3, C: IaA25R3, D: IaA22R3, E: IaA19R3 F: IaA18R3, G: IbA9G3R2, H: IdA14G1, I: IdA15G1, J: IeA11G3T3, K: IfA13G1, L: IfA16G1, M: IIcA5G3a N: IIdA13G1. Side panels show the intensity scale for the amount of antibody binding to alleles expressed by IVTT and spotted on the array. Antibody binding to the purified recombinant relatively conserved Cp17 peptide was included on the array as a positive control. Its signal intensity was higher than that of the IVTT values.

The protein encoded by the *gp60* gene is processed by the parasite into Gp40 and Gp15 proteins (Figure 6A). The region of the *gp60* gene that encodes the Gp40 protein has 3 variable domains: a SNP-based allelic family “type”; a variable number of trinucleotide repeats “subtypes”; and a repeat sequence “R” (40). In the Bangladeshi infant cohort, 15 different variants of *gp60* were identified (2 in *C*. *parvum* and 13 in *C*. *hominis*) (Figure 6A), all of which were included in the protein array (35). The *gp60* genotype of the infecting *Cryptosporidium* parasite was known in a subset of cases, and the data from the plasma collected from these children was examined to see if an allele-specific immune signal could be observed. With 1 exception, the infecting *C*. *hominis* genotype matched the Gp40 antigen variant recognized by the child’s plasma (Figure 6, B and C, and Supplemental Figure 7). The number of trinucleotide repeats in the *gp60* gene, however, did not impact antibody recognition: children infected with the IaA18R3 subtype bound equivalently to the Ia antigens on the array that had different numbers of trinucleotide repeats, including IaA27R3, IaA26R3, IaA25R3, IaA22R3, IaA19R3, and IaA18R3 (Figure 6, A and B). We concluded that humoral immunity to the variable Gp40 antigen was genotype-specific.

### Antigens associated with protection from reinfection.

**Figure 7:**
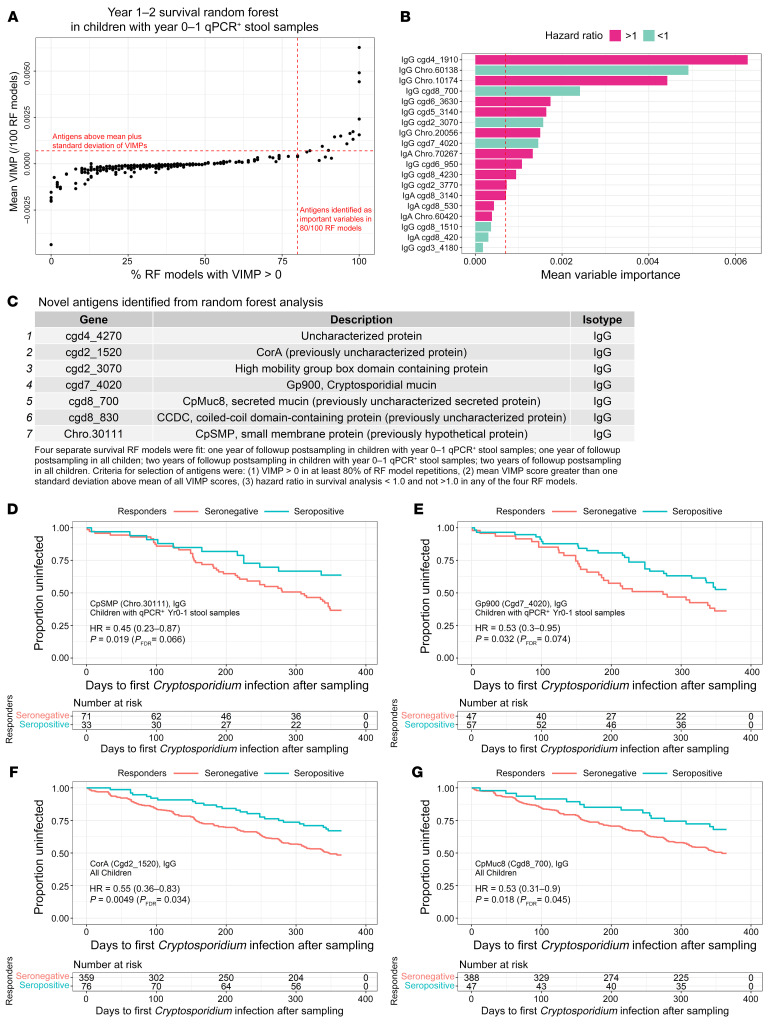
RF analysis for selection of important antigens and analysis of risk during the first year after sampling. (**A**) The scatter plot represents antigens and clinical variables ranked by VIMP scores in RF using 1,000 trees constructed per model. Models were fit to survival data during one year of follow up after sampling on seropositive and seronegative children that all previously had qPCR-confirmed *Cryptosporidium* infections. Models using the entire cohort of children and 2-year follow-up periods are shown in Supplemental Figure 8. Each model was repeated 100 times, and the VIMP score was averaged across all runs (Y-axis). For each antigen, the percentage of runs where VIMP was greater than 0 (i.e., important to the model) was calculated (X-axis). The red horizontal dashed lines represent the mean of all VIMP scores plus 1 SD. The vertical dashed red lines represent antigens with at least 80% positive VIMP scores. The upper right quadrant shows the antigens selected as important variables in the model. (**B**) The horizontal bar plot represents VIMP scores for each antigen with at least 80% positive VIMP scores. The vertical red dashed line represents the cutoff for selection of important variables (equivalent to the horizontal lines in **A**). HRs calculated in the survival analysis were shown as protective (HR < 1, teal) or not (HR > 1, magenta). (**C**) Only protective antigens with at least 80% positive VIMP scores and VIMP scores above the importance cutoff were selected for individual antigen analysis. (**D**–**G**) The Kaplan Meier plots represent the 2 most significant previously unknown antigens associated with protection in children with prior qPCR^+^ stool samples or all children, respectively, after feature selection using RF.

We tested if a delay in the time to reinfection was associated with the development of anti-*Cryptosporidium* antibodies against specific antigens. This analysis was done for the children on follow up to ages 2 and 3 years who had qPCR-verified cryptosporidiosis during their first year of life (Table 1 and Supplemental Figure 8). The analysis was also performed including all the children in the cohort (Supplemental Table 2 and Supplemental Figure 9). To minimize false discoveries as well as false exclusions, a feature selection antigen filtering step was employed using random forest (RF) models on survival data. For the RF models, the children were stratified into seropositive versus seronegative for each of the 233 IgA- and/or IgG-reactive antigens, and variables that were important to the models over 100 iterations were identified (Figure 7, A and B).

Among antigens with an average variable importance metric (VIMP) greater than 1 SD above the mean of all antigen VIMP scores and with positive VIMP scores (i.e., important to the model) in at least 80% of iterations, 7 antigens in addition to Gp60 and Cp23 had hazard ratios less than 1 (protective) in all 4 modeling groups (Figure 7C). Additional RF comparisons are shown in Supplemental Figure 8 and are included in Table 1 along with the adjustment for the number of antigens tested. Additional antigens associated with protection in survival analyses, but not by random forest, are shown in Supplemental Figure 12 and Supplemental Table 3). These were selected for evaluation in Cox proportional hazards models (Figure 7B) with adjustment for the FDR. In addition to the Gp60 and Cp23 antigens, a significant association with protection from cryptosporidiosis was observed for antibodies against the Gp900 mucin (cgd7_4020), the potential mucin CpMuc8 (cgd8_700), the putative metal ion transporter CpCorA (cgd2_1520), a small membrane protein (Chro.30111) (Figure 7D), the Gp900 mucin (cgd7_4020) (Figure 7E), the putative metal ion transporter CpCorA (cgd2_1520)(Figure 7F), the potential mucin CpMuc8 (cgd8_700)(Figure 7G), and the coiled coil domain protein CpCCDC (cgd8_830) (additional RF comparisons are shown in Supplemental Figure 8). Parenthetically, cgd8_830 seropositivity was associated with significantly lower incidence of infection, particularly during the first year of followup postsampling, but was found to be more abundant in children that ultimately were infected at the end of followup. Likewise, Chro.30111 antibody responses showed evidence of protection during followup and at the end of the first year of followup postsampling, but not at the end of 2 years of followup.

**Figure 8:**
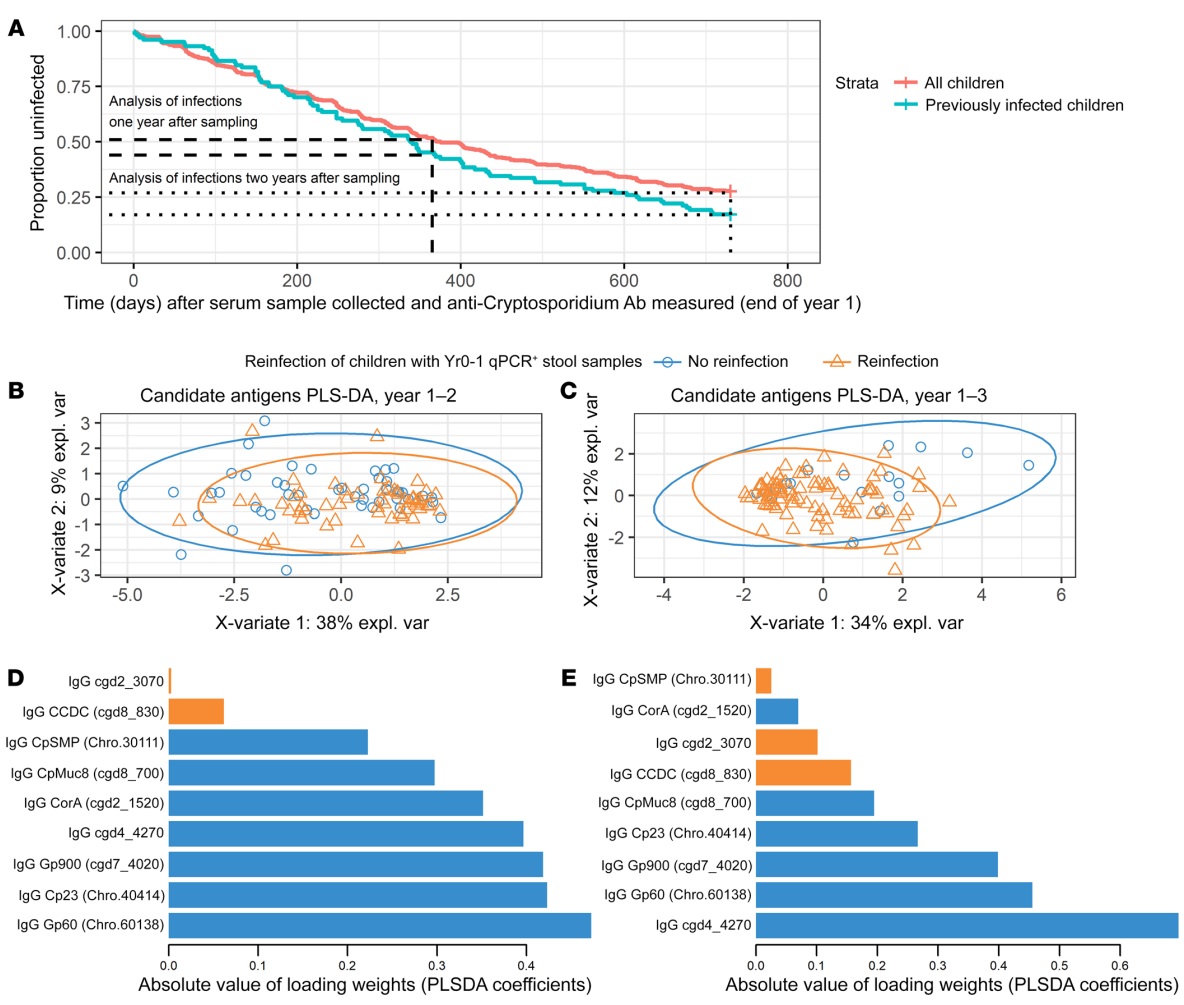
*Cryptosporidium* antigens associated with the development of a protective immune response. (**A**) The survival curve illustrates the 2 subgroups of children and 2 follow-up periods after plasma was collected (end of year 1) that were analyzed for protection. The blue line follows only children who had a qPCR-verified *Cryptosporidium* infection (subclinical or symptomatic) during the first year of life, prior to sampling plasma. The red line follows all children in the array study and includes the immunologically naive children that remained uninfected at 1 year of age as well as those known to be previously infected. Dotted and dashed lines indicate the time points (year 2 and year 3) selected for analysis of the differences between uninfected and infected groups looking specifically at the protective candidate antigens identified in Table 1. (**B** and **C**) PLS-DA on the antibody profiles of the candidate antigens shown in Table 1 associated with either reinfection or protection. Each point represents the immune profile from 1-year-old children with prior qPCR-verified *Cryptosporidium* infections who were subsequently uninfected (blue circles) or reinfected (orange triangles) during the 1-year follow-up period (**B**) or 2-year follow-up period (**C**) after plasma samples were collected. (**D** and **E**) Predictor loadings derived from the PLS-DA analysis in (**B** and **C**) are shown, respectively. Antibody targets are shown on the Y-axis, and the X-axis shows the absolute value of the loading weights (or PLS-DA regression coefficients); the absolute value was used to focus attention on the importance of each antigen in maximizing the covariance between antibodies and *Cryptosporidium* infection outcomes. Orange bars indicate antibodies more abundant in the children who subsequently had a new *Cryptosporidium* infection and blue bars indicate the antibodies more abundant in the uninfected children.

A PLS-DA regression model was then used to evaluate the relative contribution of the selected antigens in defining the latent components (“loading weights”) that maximize discrimination of children by infection status (Figure 8A). The endpoint metric was complete protection from reinfection associated with antibody levels. The analysis was performed at ages 2 (Figure 8B) and 3 (Figure 8C). The contribution of each antibody to the PLS-DA profile at ages 2 (Figure 8D) and 3 (Figure 8E) is also shown.

## Discussion

The most important discovery from this work is the identification of 7 cryptosporidial antigens to which a humoral immune response is associated with protection from reinfection. These included previously identified vaccine candidates Cp23 and Cp17 proteins; (7, 8, 34, 41) the Gp900 mucin (16, 25, 30); as well as CpMuc8, a potential mucin; CpSMP1, a small membrane protein; CpCCDC, a coiled-coil protein; and CpCorA,a potential metal transporter.

The developmental stages of *Cryptosporidium* (36) include extracellular and intracellular forms that differ in protein repertoire (37, 38, 42). We considered it a possibility that only antigens from proteins expressed at specific stages in the parasite’s lifecycle were protective. Antibodies that target the early extracellular life stages in apicomplexans may be more effective at preventing infection (31, 43). However, in our case, the protective antigens were present in more than one life stage, as assessed by mRNA transcripts (36). A drawback of using transcriptomics data is that posttranscriptional regulation may influence protein expression. We, therefore, examined the available proteomic data and found peptides from 4 of the 7 candidates in the sporozoites/oocyst proteome (37, 38). In summary, the protective antigens are not all highly expressed in sporozoites. The available information suggests that protective antigens may not be restricted to the cryptosporidial sporozoite.

While plasma IgG anti-Cp23 and Cp17 had not been previously observed to be protective in our cohort (8, 10), reanalysis of the IgG data in our study suggests that our use of the mixed models to determine the boundary of the positive response in the protein array data improved the specificity of the immunoassay results and accounted for the apparent difference in the study conclusions (Supplemental Figure 4) (7, 44).

The association observed between antibody levels and time since detection of *Cryptosporidium* infection suggests that anti-*Cryptosporidium* antibodies may be short-lived in this young age group. It is possible that multiple repeat infections are needed to generate a more durable antibody response (45, 46). In addition, a substantial proportion of the children with prior infection had largely undetectable antibody levels, similar to the larger population of immunologically naive children. Failure to generate robust humoral immunity in infants has also been observed with infection with the apicomplexan parasite *Plasmodium*, with immunity to symptomatic disease developing only after repeated exposures to the pathogen (47, 48). Also in *Plasmodium*, a short-lived nonsterile immunity is common in infants, and in this case may involve defects in antibody affinity maturation in the host germinal centers (47, 49, 50). Whether this may also play a role in depressing the antibody response to the *Cryptosporidium* parasite, and if this can be remedied by an appropriate vaccination strategy remains to be discovered.

The immune mechanisms involved in the control of the *Cryptosporidium* parasite likely involve both the innate and adaptive arms of the immune system (1). The adaptive immune response consists of both cellular and humoral immunity. Preexisting anti-*Cryptosporidium* IgG was shown to be associated with immunity in this work and earlier in experimentally infected adults (27, 34, 51). The importance of cellular immunity and, in particular, IFN-γ is also evident (52–54). It remains to be determined the contribution of these 7 antigens to cellular immune responses.

In addition to the antibodies associated with protection, we identified some anti-*Cryptosporidium* antigen-specific antibody responses that were associated with an increased risk of reinfection (Figure 7B and Supplemental Figure 9). These antibodies occurred in a distinct subset of children, and their appearance was not correlated with that of the antibodies associated with the protective immune response (Supplemental Figure 10). This observation is in line with our earlier finding that not all antigen-antibody responses are associated with protection (Figure 4). We investigated but found no association between the appearance of the antibodies targeting these nonprotective antigens and biomarkers of inflammation or of IL-4, which is involved in modulating the humoral immune response (Supplemental Figure 11). Further studies are needed to identify the mechanism that promote a protective immune response.

A limitation of this study to identify antibody responses associated with protective immunity was that not every infection was detected, despite active surveillance that included twice-weekly home visits. This coupled with the heterogeneity observed in the antibody profile among children with prior exposure, i.e., lack of antibody responses in a subset of children, likely reduced the statistical power of our analysis. Another limitation was the inability to probe the protein microarray with fecal IgA due to high background. A final limitation was that the microarray did not contain the entire proteome of the *C*. *parvum*, *C*. *hominis,* and *C*. *meleagridis* parasites. To offset this limitation, we prioritized the inclusion of the potential vaccine candidates mentioned in earlier literature (*n* = 22), as well as conserved transmembrane and secreted proteins (Supplemental Figure 3 and Supplemental Table 1) (13–33). Of the *C*. *parvum* genes with no introns, 336 of 761 ORF annotated as containing a transmembrane domain were included in the protein microarray. The remaining 425 ORFs were eliminated as being either absent in the other *Cryptosporidium* species of *C*. *meleagridis* and *C*. *hominis,* or were represented by other orthologues on the array, or because they were part of the endomembrane system, components of the mitochondria, ribosome, or nucleus. We avoided potential metabolic proteins, annotated as DNA and RNA binding, involved in one of the biosynthetic pathways of the parasite, or involved in the ubiquitin pathway. We also eliminated genes with a nonsynonymous/synonymous substitution rate over 1 and that had at least 10 nonsynonymous SNPs. Sixteen of the *C*. *parvum* ORF candidates failed at either the cloning step or during IVTT quality control. Seventy-one of the remaining ORFs that otherwise met our criteria as conserved membrane proteins could not be included on the array due to space limitations.

Strengths of this study included the intensive surveillance undertaken for cryptosporidiosis from birth to 3 years of life and the use of a proteome array that enabled the identification of previously unknown protective antigens (55). In addition to the antigens discussed, these included some antigens that, although interesting, were not statistically significant (Supplemental Figure 11). This comprehensive identification of *Cryptosporidium* antigens to which an antibody response is associated with protection represents an important step forward in vaccine design. At the same time, the fact that many children do not develop a detectable antibody response to infection, and that the antibody responses, when generated, are short lived and parasite-genotype specific may offer insight into why recurrent infections are common.

## Methods

### ORF selection for the Cryptosporidium protein microarray.

Although many *Cryptosporidium* species can infect humans, the most common human pathogens are the zoonotic *C*. *parvum* and *C*. *meleagridis* and the anthroponotic *C*. *hominis* (6). The selected *C*. *parvum* proteins included those that had been previously identified as potential vaccine candidates (Supplemental Table 1) (6, 14–34). The genome sequences of *C*. *parvum*and *C*. *hominis* are very conserved (95%–97%) and *C*. *meleagridis* is not very divergent (8.5%; *C*. *parvum* versus *C*. *meleagridis*) (55, 56) The array included 522 conserved antigens derived from proteins (*n* = 376) that contained signal peptides (*C*. *parvum*: 382 antigens; *C*. *hominis*: 155 antigens [134 derived from the *C*. *hominis* orthologues of “high value” *C*. *parvum* proteins already on the array]); and *C*. *meleagridis*: 6 antigens [also represented by other orthologues on the array] (CryptoDB Database: Release 59 (57, 58)). Due to discovery of export motifs such as PEXEL in other apicomplexan parasites, we did not restrict our antigen selections to only those with a signal peptide (59, 60). Additional antigens from proteins (*n* = 327) without a signal peptide, but which nevertheless had a transmembrane domain, were included on the array (total antigens: 457; *C*. *parvum* antigens: 365; *C*. *hominis* antigens: 90 [66 were derived from the *C*. *hominis* orthologues of *C*. *parvum* proteins already on the array]; *C*. *meleagridis* antigens: 2 [both also represented by other orthologues on the array]).

### Cryptosporidium protein microarray fabrication.

The protein microarray used in this study was produced by Antigen Discovery Inc. (ADI) (39, 61, 62). Briefly, the open reading frames selected as described above were subcloned into a T7 expression vector pXI and expressed using an in vitro the *Escherichia coli* transcription and cell free translation (IVTT) system (Rapid Translation System, Biotechrabbit). After expression the proteins were printed onto nitrocellulose-coated AVID slides (Grace Bio-Labs Inc.) using an Omni Grid Accent robotic microarray printer (Digilabs Inc.). As a positive control, the purified recombinant *Cryptosporidium* Cp17 and Cp23 peptides were also spotted onto the array (34, 63).

### Child cohort.

A total of 500 children were enrolled within 1 week of birth from the Mirpur community of Dhaka, Bangladesh from June 2014 through March 2016 (ClinicalTrials.gov, NCT02764918) (11). Infants were monitored for cryptosporidiosis through testing of diarrheal and monthly surveillance stool samples during biweekly home visits by trained field investigators. Height and weight were measured quarterly to assess child growth (11). Children who had a HAZ score under –1 were defined as ‘at risk for stunting’ and HAZ under –2 were defined as stunted (64).

### Sampling and specimen testing.

Fecal DNA was extracted from the diarrheal and monthly surveillance stools and a previously described multiplex qPCR assay that utilizes pan-*Cryptosporidium* primers and probes targeting the 18S rDNA gene was used to identify infected infants (11). In select isolates, the parasite was genotyped using standard protocols (35). Assays to measure select biomarkers of gut and systemic inflammation (CRP, sCD14, IL-1Beta, and IL-4) were performed as previously described (11). A blood sample was drawn at 18 weeks, 1 year, and then every 6 months (dbGAP Accession phs001665.v1.p1). The plasma samples from children collected at 1 year of age were diluted 1:100 and used to probe the *Cryptosporidium* proteome microarray using standard protocols (61). Samples were incubated on the arrays overnight at 4°C on a rocker, then washed and incubated with polyclonal goat anti-human IgG-Fc fragment DyLight650 (Bethyl Laboratories, A80-104D5) or Cy3 AffiniPure F(ab′)_2_ fragment of the polyclonal goat anti-human serum IgA, α chain specific (Jackson ImmunoResearch Laboratories, 109-166-011) for 1 hour at room temperature on a rocker, then washed, dried and stored in the dark until scanning. The exposed arrays were scanned, and the spot and background signal intensities (SI) were exported into R for statistical analysis (65). Spot SIs were adjusted for local background by subtraction, and values were floored to 1. Next, the data were normalized by dividing the *Cryptosporidium* protein spot values by the median of IVTT control spots (IVTT expression reactions with no *Cryptosporidium* ORFs), and values were log transformed using the base-2 logarithm. Thus, normalized data represented the log_2_ signal-to-noise ratio, where a value of 0 represents specific antibody SI equal to the background, 1.0 represented twice the background, 2.0 represented 4-fold over background, etcetera. For the purified Cp23 protein and Cp17 peptide printed on the microarrays, the data were unaffected by the IVTT background, and thus the normalization procedure was to floor the data to 1 and then transform values using the base-2 logarithm, resulting in normalized data that represent a doubling in fluorescence SI per unit increase. Thus, the scales of purified proteins and IVTT proteins differed due to the former being log-scale SI levels and the latter being log-scale signal-to-noise ratios. The results from the protein microarray are included in the Supplemental Data Set 1.

### Statistics.

*Cryptosporidium* protein responses were classified as seropositive or negative by taking the distribution of each spot individually between all samples to model negative and positive subpopulations using mixture models executed with the “normalmixEM” function in the mixtools package (66), and a seropositivity cutoff was established for each antigen as the mean and 3 SDs of the negative SI distribution. When mixture models failed to converge, a simple seropositivity cutoff of 1.0, or 2-fold over background, was applied. IgG and IgA “reactivity” for each antigen was defined as a proportion of seropositive responses, or seroprevalence, of at least 10% among the study children. Overlap in IgG and IgA responses that recognized individual antigens was assessed using the VennDiagram package in R (67). Antibody breadth scores were calculated as the sum of seropositive responses per individual. Normalized SI and antibody breadth scores were visualized using the ComplexHeatmap package (68). Associations between normalized SI and protein features such as life cycle stage and signal peptides were analyzed using multivariable negative binomial regression for seropositivity classifications and ordinary least squares regression for normalized SI. Univariate groupwise analysis was performed using *t* tests. Correlations were assessed using Pearson’s correlation coefficient. Unsupervised data reduction and trends was analyzed using tSNE, using a perplexity parameter of 40, 1,000 iterations, and a theta of 0.0 after testing varying parameters for shape of the data (69, 70). To test differences in profile-wide normalized SI and to control for multiple measurements per subject, linear mixed effects regression (LMER) was performed using the lme4 package (71), allowing for random intercepts at the subject and antigen level. *P* values for LMER models were obtained by ANOVA between full models and null variable models. Supervised data reduction and multi-antigen analysis was performed using PLS-DA using the mixOmics package (72). PLS-DA results were visualized using plots of PLS-DA scores of first and second latent variables for trends discriminating groups and by plotting the loading weights (i.e. PLS-DA regression coefficients) to highlight the importance of the variables used to define the latent components that maximize the covariance between antibody data and *Cryptosporidium* infection endpoints (infection prior to sampling and infection 1 and 2 years after sampling). Analysis of antibody breadth was performed using negative binomial regression.

Analysis of seropositivity to an antigen and association with risk of infection or reinfection — only among children with infections prior to 1 year of age — during the 1-year and 2-year follow-up periods following sample collection was performed using Cox proportional hazards models adjusted by the number of episodes a child had; HAZ score at birth; mother’s age, BMI, and education level; household income and expenses; principal source of household drinking water; the water treatment method routinely used by the household; and proximity to one of the Dhaka water drainage channels. The 2 a priori vaccine candidate antigens Gp60 — containing the Cp17 peptide — and Cp23 were analyzed without correction for the FDR (73).

To control for false discovery due to the multiple comparisons made, antibody responses were modeled using random forest (“RF”) with survival data using the “randomForestSRC” and “ggRandomForests” packages to perform feature selection (74). All reactive antigens were included in each of 4 RF models: 1 year follow-up postsampling among (a) all children or (b) only children with previous infections, and (c) 2 year follow-up among (c) all children or (d) only children with previous infections. Each model performed 1,000 decision trees while allowing deterministic splitting (nsplit = 0) and computation of variable importance (VIMP) using permutation. Each model was repeated 100 times, and VIMP scores were computed for each repeat and then averaged for each antigen target. VIMP scores that were greater than 0 in at least 80% of repeats (80/100 RF models) were considered “potentially important variables” for the RF models. Further, a VIMP score cutoff was calculated as 1 SD above the mean of all VIMP scores returned from the 100 RF model iterations. The survival analysis identified which antibodies were associated with a HR below 1 (protective response) versus HRs greater than 1 (increased susceptibility). The criteria determined for selection of a minimal number of important variables were: (a) positive VIMP scores in at least 80% of repeats and average VIMP scores above the cutoff, and (b) seropositivity HRs below 1 in all models. The selected features were used in Cox models, reporting both raw *P* values and *P* values adjusted for the FDR. Analysis of Gp40/15 (aka Gp60) sequence variation and antibody associations was performed using the Prism 9 computer program (GraphPad).

### Study approval.

The study was approved by the Ethical and Research Review Committees of the International Centre for Diarrhoeal Disease Research, Bangladesh (PR-13092) and the IRB of the University of Virginia (IRB# 20388). The ClinicalTrials.gov identifier is NCT02764918. Informed written consent was obtained from the parents or guardians for the participation of their child in the study.

### Data availability.

Select clinical metadata for this study is available on the NCBI’s dbGaP under accession number phs001665.v2.p1. The data for this study were collected as a substudy of dbGaP phs001475.v2.p1.

## Author contributions

Drafting of the manuscript was performed by CAG and JJC. The method used to assign initial authorship order was by mutual agreement. All authors edited and approved the final manuscript. CAG, WAP, and RH conceived of the analysis plan and JJC performed the bioinformatic analyses. JZM assisted in the statistical analysis. CAG designed and JJC, JVP, AT AO, and ADS built the array and analyzed samples. WAP, RH, and AF founded the birth cohort and directed the study. Field work and data collection at the International Centre for Diarrhoeal Disease Research, Bangladesh (icddr,b) were performed by MA and MK, with supervision from AF and RH.

## Supplementary Material

Supplemental data

Supplemental data set 1

Supporting data values

## Figures and Tables

**Table 1:**
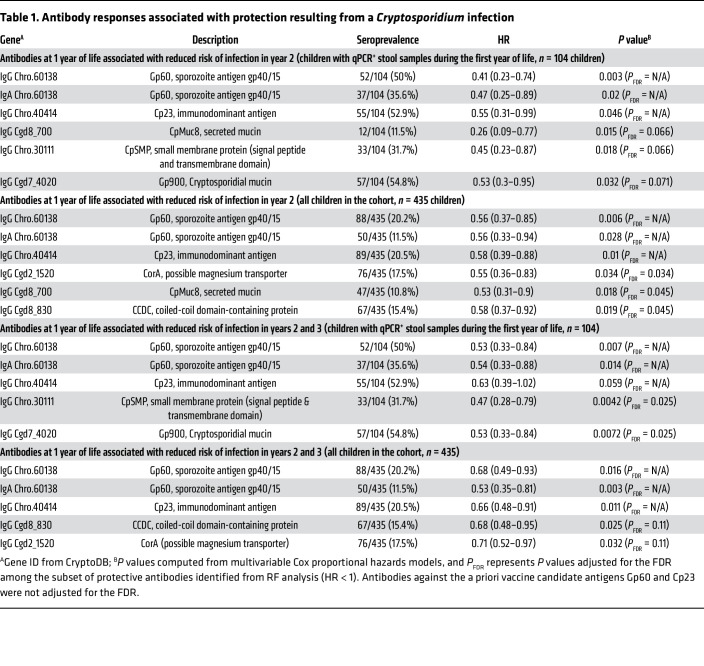
Antibody responses associated with protection resulting from a *Cryptosporidium* infection

## Version 1 06/22/2023

In-Press Preview

## Version 2 08/15/2023

Electronic publication
