# Supplementary material for: Specific Cryptosporidium antigens associate with reinfection immunity and protection from cryptosporidiosis
Source: J Clin Invest. 2023 Aug 15;133(16):e166814. doi: 10.1172/JCI166814 (PMC10425216; doi:10.1172/JCI166814)

Supplemental Table 1 Potential vaccine candidates selected for inclusion in the *Cryptosporidium* protein array

Name*	Gene ID (CryptoDB)			Reference	IgG prevalence	IgA prevalence
CpGP900	cgd7_4020	Chro.70447	CmeUKMEL1_03740	(30, 28)	(see Table 1)	
gp15/gp40	cgd6_1080	Chro.60138	CmeUKMEL1_04910	(30)(36) (27) (26) (25)	(see Table 1)	
cp23	cgd4_3620	Chro.40414	CmeUKMEL1_08080	(29)	(see Table 1)	
cp20	cgd7_1280	Chro.70153	CmeUKMEL1_10725	(30)	72	55
P2	Cgd2_130	Chro.20020	CmeUKMEL1_09185	(32, 33)	5	10
CpMuc4	cgd2_420	Chro.20049	CmeUKMEL1_09045	(24)	41	15
CpMuc5	cgd2_430	Chro.20050	CmeUKMEL1_09040		18	13
UDP-GlcNAc	cgd7_1830	Chro.70213	CmeUKMEL1_04435	(23)	13	6
PFP	cgd3_1400	Chro.30172	CmeUKMEL1_02790		13	3
Apyrase	cgd6_1570	Chro.60194	CmeUKMEL1_04670	(20)	3	2
profilin	cgd3_1570	Chro.30189	CmeUKMEL1_02865		12	4
cp15	cgd4_2330	Chro.40263	CmeUKMEL1_02290		20	3
cp21	cgd2_2570	Chro.20274	CmeUKMEL1_16905	(21)	20	11
cp47	cgd6_1590	Chro.60196	CmeUKMEL1_04660		5	3
EF-1α	cgd6_3990	Chro.60459	CmeUKMEL1_10355	(19)	20	15
Cp2	cgd6_5410	Chro.60623	CmeUKMEL1_05715	(18)	36	3
Cpa135/ CpSUB	cgd7_1730	Chro.70203	CmeUKMEL1_04490	(17) (14)	20	6
TRAP-C1	cgd5_3420	Chro.50029	CmeUKMEL1_16195	(16)	45	27
CpMIC1/TSP1	cgd6_780	Chro.60102	CmeUKMEL1_05055	(13)	13	4
LCFAS	cgd4_3400	Chro.40386	CmeUKMEL1_07975	(74)	9	117
P30	cgd6_2330	Chro.60272	CmeUKMEL1_12950	(75)	11	11

CSL	cgd3_2340<br>cgd3_930	GY17_00002458<br>GY17_00002617	CmeUKMEL1_12035<br>CmeUKMEL1_02560	(76)	0	1

*Table derived from Bouzid et al (31)

Supplemental Figure 1

Frequency of new *Cryptosporidium* infections over the first three years of life. Positive samples (Cq values <40) were grouped into one infection if collected at < 65-day intervals. The day when the first positive sample was collected was used for tabulation purposes and reported as the child's age in days. The number of new infections (diarrheal or sub-clinical) in each 30.4 days window is displayed on the Y axis. On the X axis is the child's age

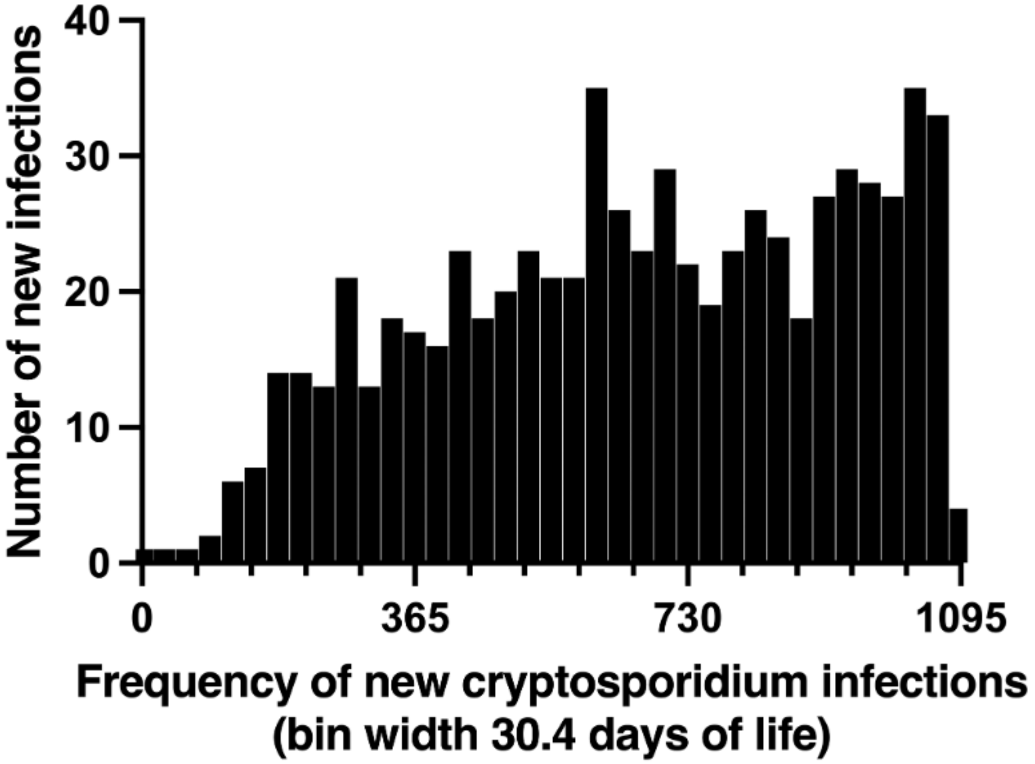

Supplemental Figure 2 **Final Cryptosporidium Protein Microarray Design**

The array included 522 conserved antigens derived from proteins (n=376) that contained a signal peptides (*C. parvum*: antigens 382; *C. hominis*: 155 antigens (134 derived from the *C. hominis* orthologues of "high value" *C. parvum* proteins already on the array); *C. meleagridis*: 6 antigens (also represented by other orthologues on the array) Additional antigens from proteins (n=327) without a signal peptide, but which nevertheless had a transmembrane domain, were included on the array (total antigens: 457; *C. parvum* antigens: 365 ; *C. hominis* antigens: 90 (66 were derived from the *C. hominis* orthologues of *C. parvum* proteins already on the array); *C. meleagridis* 2 (both also represented by other orthologues on the array). The *Cryptosporidium* parasites have a complex life cycle. Within the host life-stages are either extracellular or intracellular, and differ in their protein repertoire

Proteins expressed in the sporozoite/oocyst life stages *Cryptosporidium* proteome (mass spectrometry data) number of proteins represented on array: 451; reactive proteins: IgG: n=64; IgA: n=28) (38, 77). Differentially expressed transcripts were included in the array design although antigens may only be translated and interact with the host immune system in the successive life stages (41, 78).

Transcriptional changes in C. parvum oocyst/sporozoites: total number of proteins represented on array: 409; reactive antigens: IgG: n=34; IgA: n=22

Transcriptional data Meront/trophozoites enriched (24h after infection) (total number of proteins represented on array: 248; reactive antigens: IgG: n= 29; IgA: n= 29) Merozoite enriched (48h after infection) (total number of proteins represented on array: 548; reactive antigens: IgG: n= 62; IgA: n= 39)

Gametocytes (in vivo and in vitro female gamonts)(37) (total number of proteins represented on array: 544; reactive antigens: IgG: n= 62; IgA: n= 42).

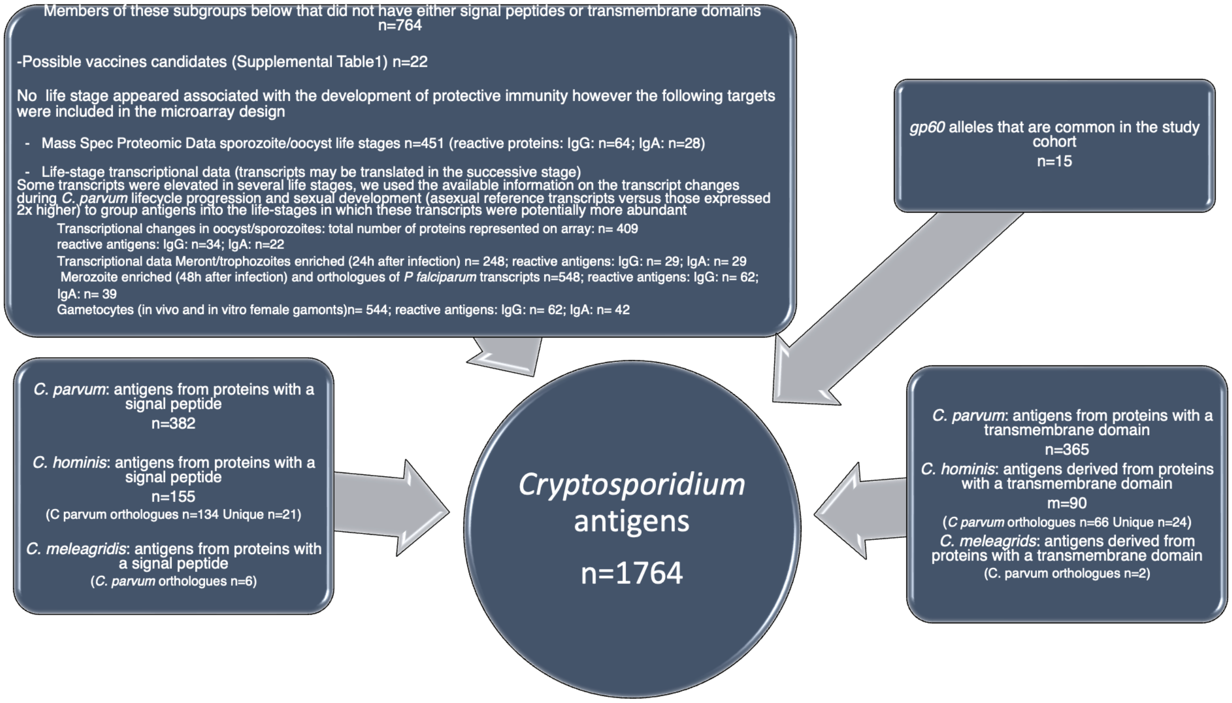
Members of these subgroups below that did not have either signal peptides or transmembrane domains
n=764
-Possible vaccines candidates (Supplemental Table1) n=22
No life stage appeared associated with the development of protective immunity however the following targets were included in the microarray design
- Mass Spec Proteomic Data sporozoite/oocyst life stages n=451 (reactive proteins: IgG: n=64; IgA: n=28)
- Life-stage transcriptional data (transcripts may be translated in the successive stage)
Some transcripts were elevated in several life stages, we used the available information on the transcript changes during C. parvum lifecycle progression and sexual development (asexual reference transcripts versus those expressed 2x higher) to group antigens into the life-stages in which these transcripts were potentially more abundant
Transcriptional changes in oocyst/sporozoites: total number of proteins represented on array: n= 409
reactive antigens: IgG: n=34; IgA: n=22
Transcriptional data Meront/trophozoites enriched (24h after infection) n= 248; reactive antigens: IgG: n= 29; IgA: n= 29
Merozoite enriched (48h after infection) and orthologues of P falciparum transcripts n=548; reactive antigens: IgG: n= 62; IgA: n= 39
Gametocytes (in vivo and in vitro female gamonts)n= 544; reactive antigens: IgG: n= 62; IgA: n= 42
gp60 alleles that are common in the study cohort
n=15
C. parvum: antigens from proteins with a signal peptide
n=382
C. hominis: antigens from proteins with a signal peptide
n=155
(C parvum orthologues n=134 Unique n=21)
C. meleagridis: antigens from proteins with a signal peptide
(C. parvum orthologues n=6)
Cryptosporidium antigens
n=1764
C. parvum: antigens from proteins with a transmembrane domain
n=365
C. hominis: antigens derived from proteins with a transmembrane domain
m=90
(C parvum orthologues n=66 Unique n=24)
C. meleagrids: antigens derived from proteins with a transmembrane domain
(C. parvum orthologues n=2)

Supplemental Figure 3 **Selection criteria for *C. parvum* antigens from proteins containing either a Signal Peptide and/or a Transmembrane Domain.** The final array included 382 conserved *C .parvum* antigens from proteins that contained a signal peptides. An additional 365 *C. parvum* antigens included on the array were from proteins without a signal peptide, but which nevertheless had a transmembrane domain. Due to both the technical challenges and the limitations on space on the array 71 of the proteins meeting these criteria were not included on the array

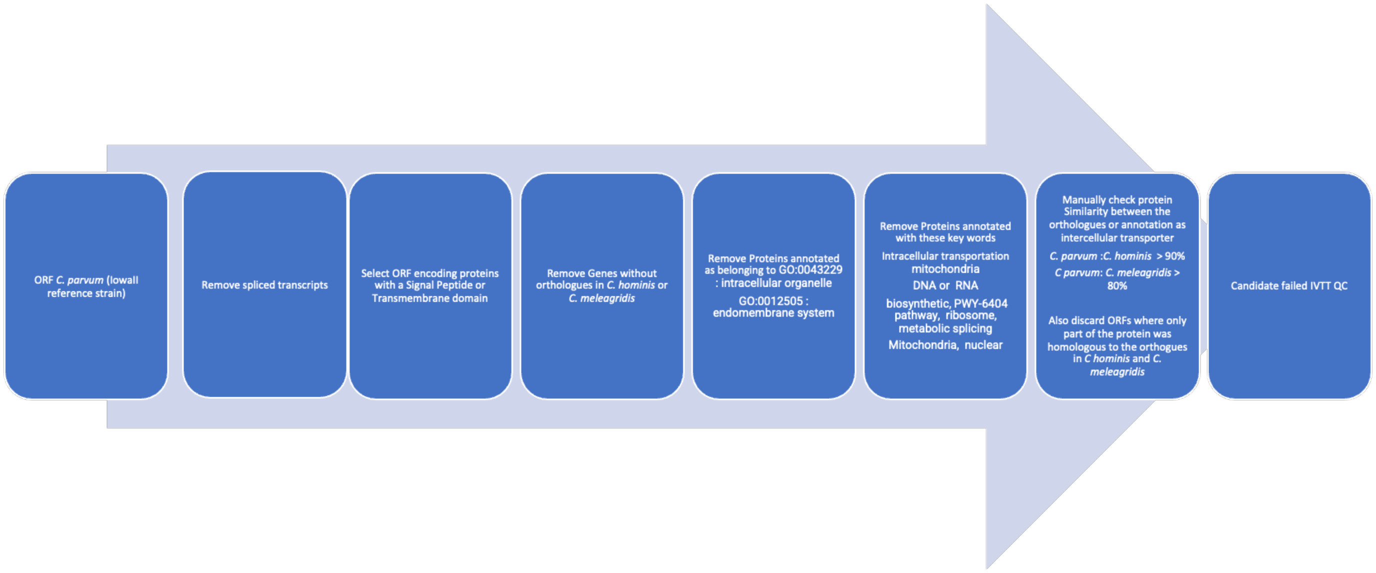

Supplemental Figure 4. Normalization of the signal from the IgG antibodies binding to the *C. hominis* antigen encoded by the *gp60* gene. A) The density plot shows the distribution of the array normalized fluorescence signal intensity (SI) values of each antigen on the X-axis. Red line: Gaussian distribution of negative signals determined by mixture model; Green line: Gaussian distribution of positive signals determined by mixture model; Blue line: antigen specific seropositivity cut-off value determined using the mean plus 3 standard deviations of the background (red line); Yellow line: high intensity signal representing signals 4-fold higher than the entire array background. B) The ROC graph compares the ability of different background cut-off values to predict which children had been previously infected (fecal DNA positive using a diagnostic qPCR assay). Dotted lines indicate the performance of the threshold cut-off values predicted by mixture model (Mx-Mdl) and median signal intensity (median).

A

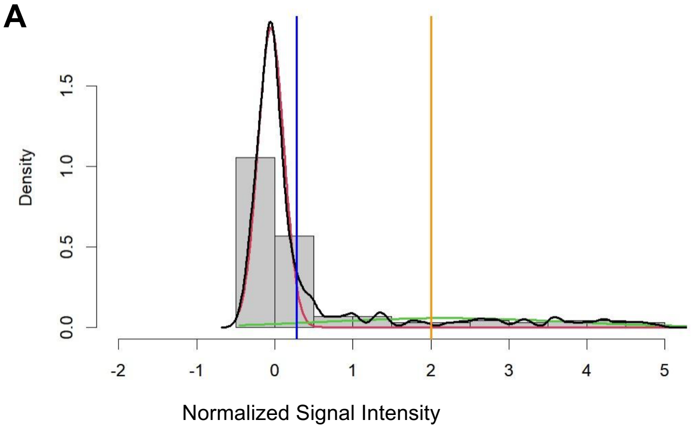

B

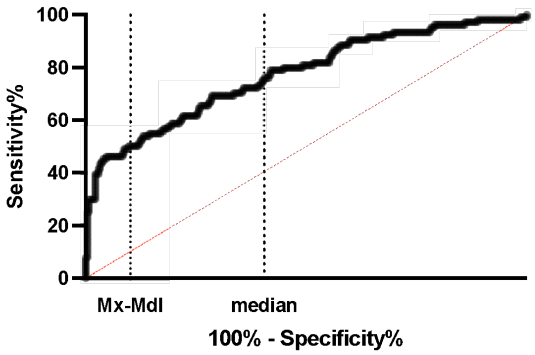

Supplemental Figure 5. **Correlation between and within IgG and IgA responses**. The correlograms show Spearman's correlation coefficients on a color scale for antibody responses between reactive Cryptosporidium antigens. Antigens are ordered using hierarchical clustering. The color scale for Spearman's correlation coefficients is located below each correlogram with red values indicating negative correlation and blue values indicating positive correlations. A) antigens reactive against IgG (n=176); B) antigens reactive against IgA (n=93); C) antigens reactive against both IgG and IgA (n=36).

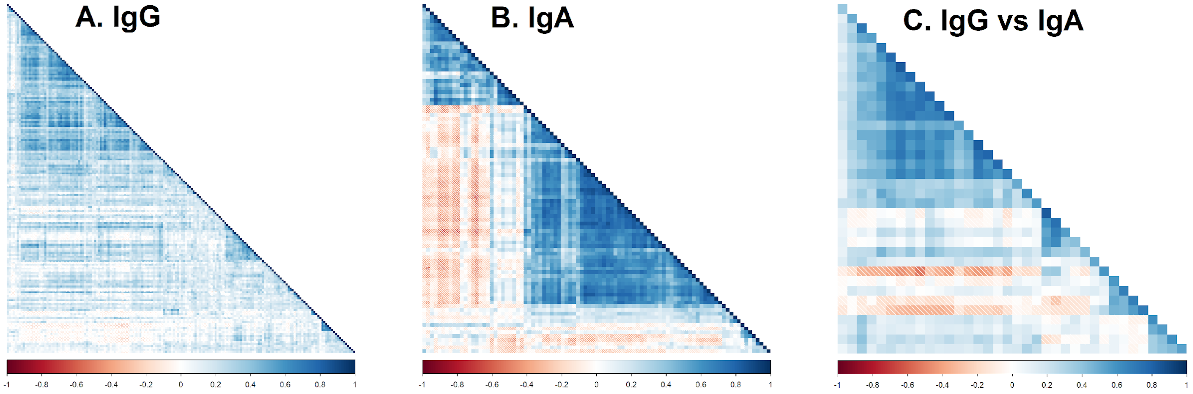

Supplemental Figure 6 **T-SNE plot of antigen responsiveness overlaid with biomarkers of immune function**. A) The overlaid color indicates child HAZ score at the end of three years as described in the figure legend. Square boxes indicated children with cryptosporidiosis. The HAZ score was not linked to any particular immunological profile. Correlation was also not observed between the immune profile and any of the biomarkers of inflammation measured in the child's plasma at one year of age B) sCD14 C) IL-1b D) CRP or E) IL-4 the biomarker of the activation of the subpopulation of T-cells which are the most biologically active helper cells for B-cells (values indicated by the appropriate figure legend).

A

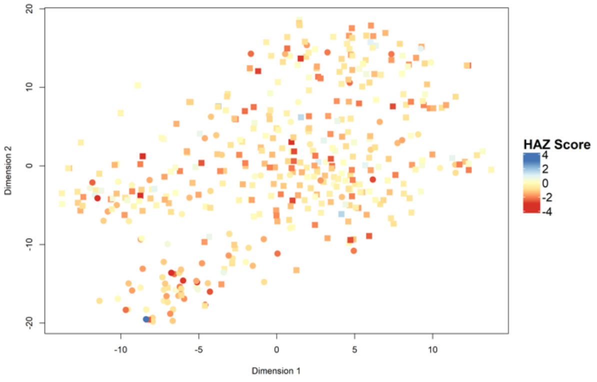

B

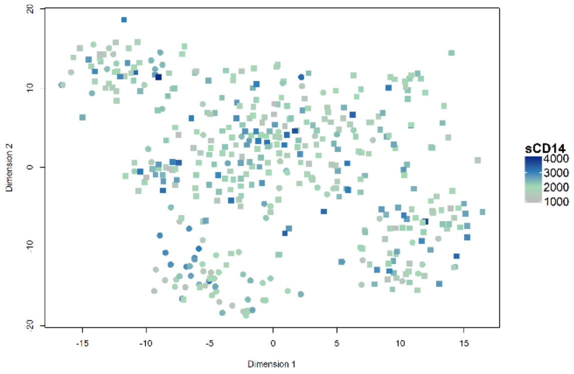

C

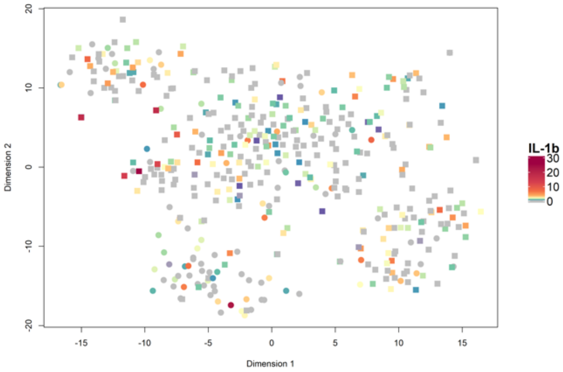

D

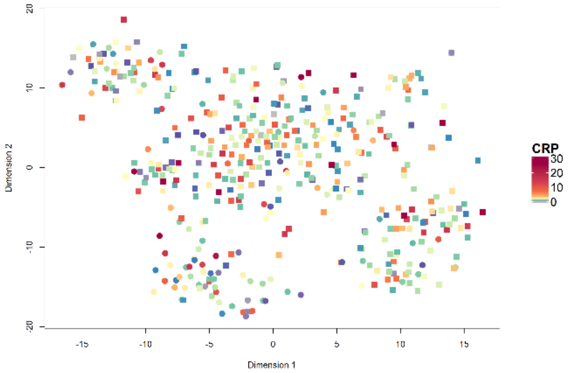

E

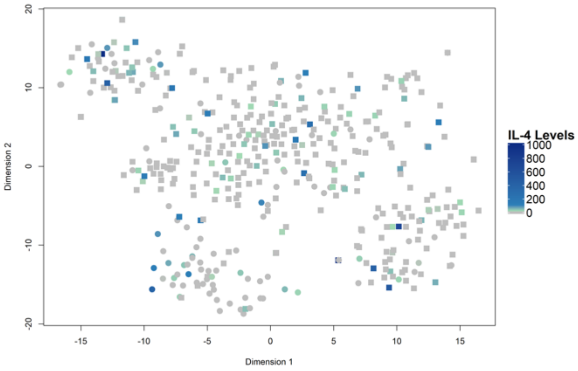
IL-4 Levels
1000
800
600
400
200
0
Dimension 2
Dimension 1

Supplemental Figure 7

Discordant results evidenced by the IVTT spot on the array. Antibody signals are shown for the antigen encoded by the IbA9G3a allele of the *gp60* gene. The left Y-axis shows the signals obtained from the IgA antibodies (blue points). The right Y-axis shows the signals obtained with the IgG antibodies (black spots). The X-axis shows the *gp60* genotypes of the parasites known to have infected the child prior to blood collection (IbA9G3a genotype in bold with*): 1-8 IaA18R3; 9; IaA19R3; 10-16 IaA25R3; 17-20 IbA9G3R28; 21-29 IdA15G1; 30 IfA13G1; 31-34 *C. parvum* IIdA15G1R1.

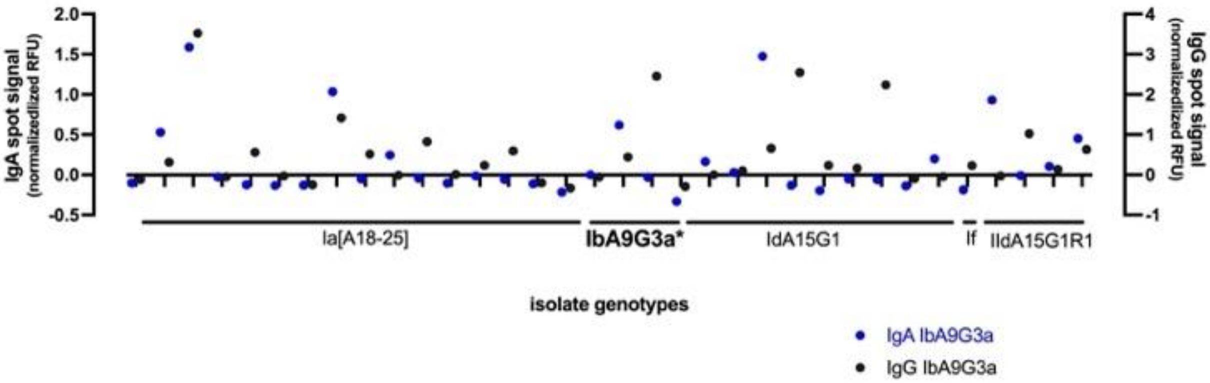

Supplemental Figure 8. Random Forest analysis for selection of important antigen variables and analysis of risk. A, C, E) The scatter plots represents antigens and clinical variables ranked by variable importance ("VIMP") scores in random forest ("RF") using 1,000 trees constructed per model. Models were fit to survival data during (A) one year of follow-up after sampling on all seropositive and seronegative children in the cohort, (C) two years of follow-up in children that had all been previously infected with *Cryptosporidium*, and (E) two years of follow-up in all children in the cohort. Each model was repeated 100 times, and the VIMP score was averaged across all runs. For each antigen, the percentage of runs where VIMP was greater than 0 (i.e., important to the model) was calculated. The red horizontal dashed lines represent the mean of all VIMP scores plus one standard deviation. The vertical dashed red lines represent antigens with at least 80% positive VIMP scores. The upper right quadrants shows the antigens selected as important variables in the model. B, D, F) The horizontal bar plots represents VIMP scores for each variable with at least 80% positive VIMP scores. The vertical red dashed lines represents the cutoff for selection of important variables (equivalent to the horizontal lines in A, C, E, respectively). Hazard ratios calculated in the survival analysis were shown as protective (HR < 1) or not (HR > 1). Only protective antigens with at least 80% positive VIMP scores and VIMP scores above the importance cutoff were selected for specific antigen analysis (Figure 7C).

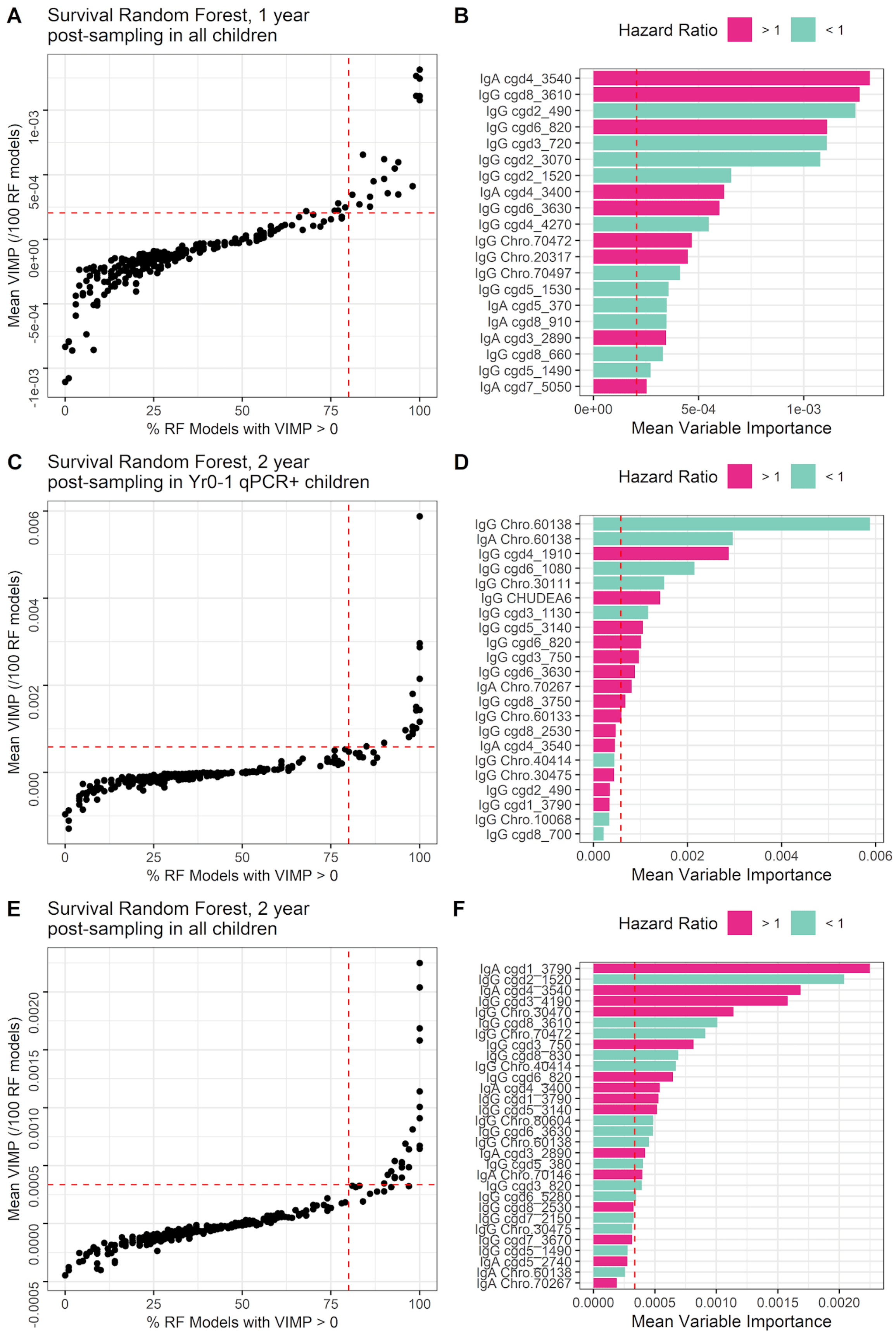
A
Survival Random Forest, 1 year post-sampling in all children
Mean VIMP (/100 RF models)
-1e-03
-5e-04
0e+00
5e-04
1e-03
0
25
50
75
100
% RF Models with VIMP > 0
B
Hazard Ratio
> 1
< 1
IgA cgd4_3540
IgG cgd8_3610
IgG cgd2_490
IgG cgd6_820
IgG cgd3_720
IgG cgd2_3070
IgG cgd2_1520
IgA cgd4_3400
IgG cgd6_3630
IgG cgd4_4270
IgG Chro.70472
IgG Chro.20317
IgG Chro.70497
IgG cgd5_1530
IgA cgd5_370
IgA cgd8_910
IgA cgd3_2890
IgG cgd8_660
IgG cgd5_1490
IgA cgd7_5050
0e+00
5e-04
1e-03
Mean Variable Importance
C
Survival Random Forest, 2 year post-sampling in Yr0-1 qPCR+ children
Mean VIMP (/100 RF models)
0.000
0.002
0.004
0.006
0
25
50
75
100
% RF Models with VIMP > 0
D
Hazard Ratio
> 1
< 1
IgG Chro.60138
IgA Chro.60138
IgG cgd4_1910
IgG cgd6_1080
IgG Chro.30111
IgG CHUDEA6
IgG cgd3_1130
IgG cgd5_3140
IgG cgd6_820
IgG cgd3_750
IgG cgd6_3630
IgA Chro.70267
IgG cgd8_3750
IgG Chro.60133
IgG cgd8_2530
IgA cgd4_3540
IgG Chro.40414
IgG Chro.30475
IgG cgd2_490
IgG cgd1_3790
IgG Chro.10068
IgG cgd8_700
0.000
0.002
0.004
0.006
Mean Variable Importance
E
Survival Random Forest, 2 year post-sampling in all children
Mean VIMP (/100 RF models)
-0.0005
0.0000
0.0005
0.0010
0.0015
0.0020
0
25
50
75
100
% RF Models with VIMP > 0
F
Hazard Ratio
> 1
< 1
IgA cgd1_3790
IgG cgd2_1520
IgA cgd4_3540
IgG cgd3_4190
IgG Chro.30470
IgG cgd8_3610
IgG Chro.70472
IgG cgd3_750
IgG cgd8_830
IgG Chro.40414
IgG cgd6_820
IgA cgd4_3400
IgG cgd1_3790
IgG cgd5_3140
IgG Chro.80604
IgG cgd6_3630
IgG Chro.60138
IgA cgd3_2890
IgG cgd5_380
IgA Chro.70146
IgG cgd3_820
IgG cgd6_5280
IgG cgd8_2530
IgG cgd7_2150
IgG Chro.30475
IgG cgd7_3670
IgG cgd5_1490
IgA cgd5_2740
IgA Chro.60138
IgA Chro.70267
0.0000
0.0005
0.0010
0.0015
0.0020
Mean Variable Importance

Supplemental Figure 9. Survival analysis of all antibodies against all reactive antigens. The volcano plots display the log hazard ratio ("HR") of multivariable Cox proportional hazards models of each reactive *Cryptosporidium* antigen (IgG or IgA seropositive vs. seronegative antibody responses) associated with time until *Cryptosporidium* infection (All Children) or reinfection (among subset of children with qPCR+ stool samples collected during the first year of life; "Yr0-1 qPCR+" Children) during the 1 year or 2 year follow-up periods after sampling at 1 year of age. Points represent antigens, and triangles represent antigens that have been identified as important variables associated with risk of infection in random forest analysis for each of the four survival analyses: (A) 1 year follow-up in previously infected children, (B) 1 year follow-up in all children in the cohort, (C) 2 year follow-up in previously infected children, and (D) 2 year follow-up in all children in the cohort. Values below 0 represent a reduction in risk (HR < 1), whereas values above 0 represent an increase in risk with seropositive antibody responses (HR > 1). The y-axis shows -log10 p-values from the Cox models adjusted for demographic and environmental variables. The horizontal red line represents a raw p-value of 0.05. The gene IDs of the proteins identified in each respective random forest analysis, as well as proteins that had raw p-values < 0.05, are labeled next to respective points. Among the antigens not identified in random forest analysis, no p-values remained significant after correction for the false discovery rate when adjusting for all a comparisons.

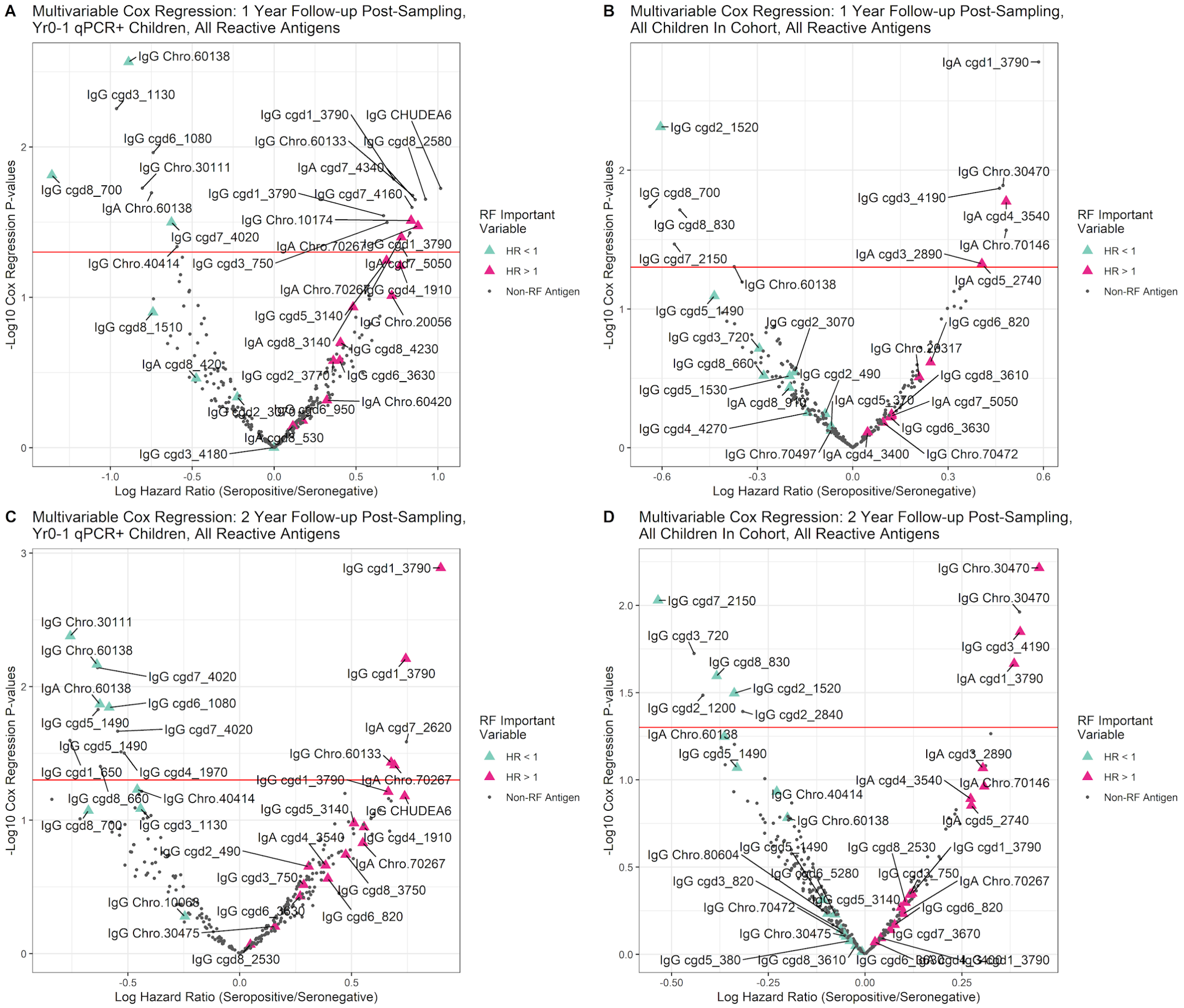
A Multivariable Cox Regression: 1 Year Follow-up Post-Sampling, Yr0-1 qPCR+ Children, All Reactive Antigens
B Multivariable Cox Regression: 1 Year Follow-up Post-Sampling, All Children In Cohort, All Reactive Antigens
C Multivariable Cox Regression: 2 Year Follow-up Post-Sampling, Yr0-1 qPCR+ Children, All Reactive Antigens
D Multivariable Cox Regression: 2 Year Follow-up Post-Sampling, All Children In Cohort, All Reactive Antigens
-Log10 Cox Regression P-values
Log Hazard Ratio (Seropositive/Seronegative)
RF Important Variable
HR < 1
HR > 1
Non-RF Antigen

Supplemental Table 2. Other antibody responses potentially associated with protection from *Cryptosporidium* reinfection

Children with qPCR-verified *Cryptosporidium* infection prior to sampling at 1 yr				Risk Reinfection 1 Yr.		Risk Reinfection 2 Yr.	
Gene*	Species	Description	Seroprevalence	IgA	IgG	IgA	IgG
cgd4_1970	*parvum*	Un-characterized trans membrane Protein	IgA: 34.6% [36/104]; IgG: 60.6% [63/104]	β=0.56 [0.3-1.05] P=0.071 (P_{FDR}=0.92)	β=0.57 [0.32-1.01] P=0.054 (P_{FDR}=0.56)	N.S.	β=0.6 [0.37-0.96] P=0.031 (P_{FDR}=0.5)
cgd5_1490	*parvum*	Un-characterized secreted Protein	IgA: 6.7% [7/104]; IgG: 29.8% [31/104]	N.S.	N.S.	N.S.	β=0.53 [0.32-0.88] P=0.015 (P_{FDR}=0.37)
cgd6_4870	*parvum*	Un-characterized protein	IgA: 9.6% [10/104]; IgG: 1.9% [2/104]	N.S.	N.S.	β=0.43 [0.17-1.07] P=0.07 (P_{FDR}=0.62)	N.S.
cgd8_3520	*parvum*	secreted protein with cysteine rich repeats and a mucin like threonine rich repeat, signal peptide	IgA: 3.8% [4/104]; IgG: 49% [51/104]	N.S.	N.S.	N.S.	β=0.65 [0.41-1.02] P=0.061 (P_{FDR}=0.61)
cgd3_1130	*parvum*	Un-characterized protein, TM domain	IgA: 12.5% [13/104]; IgG: 31.7% [33/104]	N.S.	β=0.38 [0.19-0.75] P=0.006 (P_{FDR}=0.4)	N.S.	N.S.
cgd1_650	*parvum*	Un-characterized secreted Protein (CpLSP	IgA: 10.6% [11/104]; IgG: 21.2% [22/104]	N.S.	N.S.	N.S.	β=0.47 [0.24-0.91] P=0.025 (P_{FDR}=0.49)

		gene family)					
cgd8_6 60	*parvum*	Un-characterized protein	IgA: 6.7% [7/104]; IgG: 23.1% [24/104]	N.S.	N.S.	N.S.	β=0.54 [0.3-0.97] P=0.04 (P_{FDR}=0.54)

Analysis using only the 104 children in the cohort known to be infected prior to year one by qPCR of stool samples. *Gene ID from CryptoDB; N.S.: Not significant; FDR: false discovery rate.

Supplemental Table 3. Other antibody responses potentially associated with protection from *Cryptosporidium* infection among all children in the cohort

All children				Risk Infection 1 Yr.		Risk Infection 2 Yr.	
Gene*	Species	Description	Seroprevalence	IgA	IgG	IgA	IgG
cgd4_1970	*parvum*	Uncharacterized transmembrane Protein	IgA: 13.6% [59/435]; IgG: 22.1% [96/435]	N.S.	N.S.	N.S.	β=0.63 [0.45-0.89] P=0.008 (PFDR=0.36)
cgd5_1490	*parvum*	Uncharacterized Secreted Protein	IgA: 2.8% [12/435]; IgG: 11.5% [50/435]	N.S.	β=0.57 [0.34-0.95] P=0.03 (P_{FDR}=0.58)	N.S.	β=0.61 [0.41-0.91] P=0.016 (P_{FDR}=0.36)
cgd6_4870	*parvum*	Uncharacterized protein	IgA: 10.3% [45/435]; IgG: 0.7% [3/435]	N.S.	N.S.	β=0.69 [0.46-1.04] P=0.073 (P_{FDR}=0.99)	N.S.
cgd7_2150**	*parvum*	transmembrane protein	IgA: 2.1% [9/435]; IgG: 11.3% [49/435]	N.S.	β=0.57 [0.34-0.96] P=0.034 (P_{FDR}=0.59)	N.S.	β=0.58 [0.39-0.88] P=0.009 (P_{FDR}=0.36)
cgd8_3520	*parvum*	secreted protein with cysteine rich repeats and a mucin like threonine rich repeat, signal peptide	IgA: 1.6% [7/435]; IgG: 20% [87/435]	N.S.	N.S.	N.S.	β=0.71 [0.52-0.98] P=0.04 (P_{FDR}=0.5)
cgd3_720**	*parvum*	very large probable mucin, 11700 aa long protein with signal peptide and pronounced Thr repeat (308 aa long)	IgA: 4.8% [21/435]; IgG: 14% [61/435]	N.S.	N.S.	N.S.	β=0.65 [0.45-0.94] P=0.023 (P_{FDR}=0.39)

cgd2_1200**	*parvum*	Ubiquitin domain-containing Steroid reductase	IgA: 23.7% [103/435]; IgG: 11.7% [51/435]	N.S.	N.S.	N.S.	β=0.65 [0.44-0.95] P=0.026 (P_{FDR}=0.39)

Analysis using all the children in the cohort (n=435). *Gene ID from CryptoDB; N.S.: Not significant; FDR: false discovery rate.

Supplemental Figure 10. Correlation between protective antibodies identified in random forest analysis and antibodies associated with higher risk of reinfection. The correlogram shows the correlation between signals for antigens with IgG responses associated with protection (hazard ratio < 1) and those associated with increased risk of infection (hazard ratio > 1) that were identified as important variables in random forest analysis. Protective antibodies: Gp60, Cp23, Gp900, CpSMP. Antibodies associated with increased risk: cgd4_1910, Chro.10174, cgd8_3610 and cgd3_4190. The X-axis shows normalized IgG signals for the antigen above the column, and the Y-axis shows the normalized IgG signals for the antigen to the right of the row. Data points are colored by children that had qPCR+ stool samples during the first year of life (Yr0-1 qPCR+, purple) and those without qPCR+ stool samples (Yr0-1 qPCR-, green). A smooth line is shown for all of the samples in red.

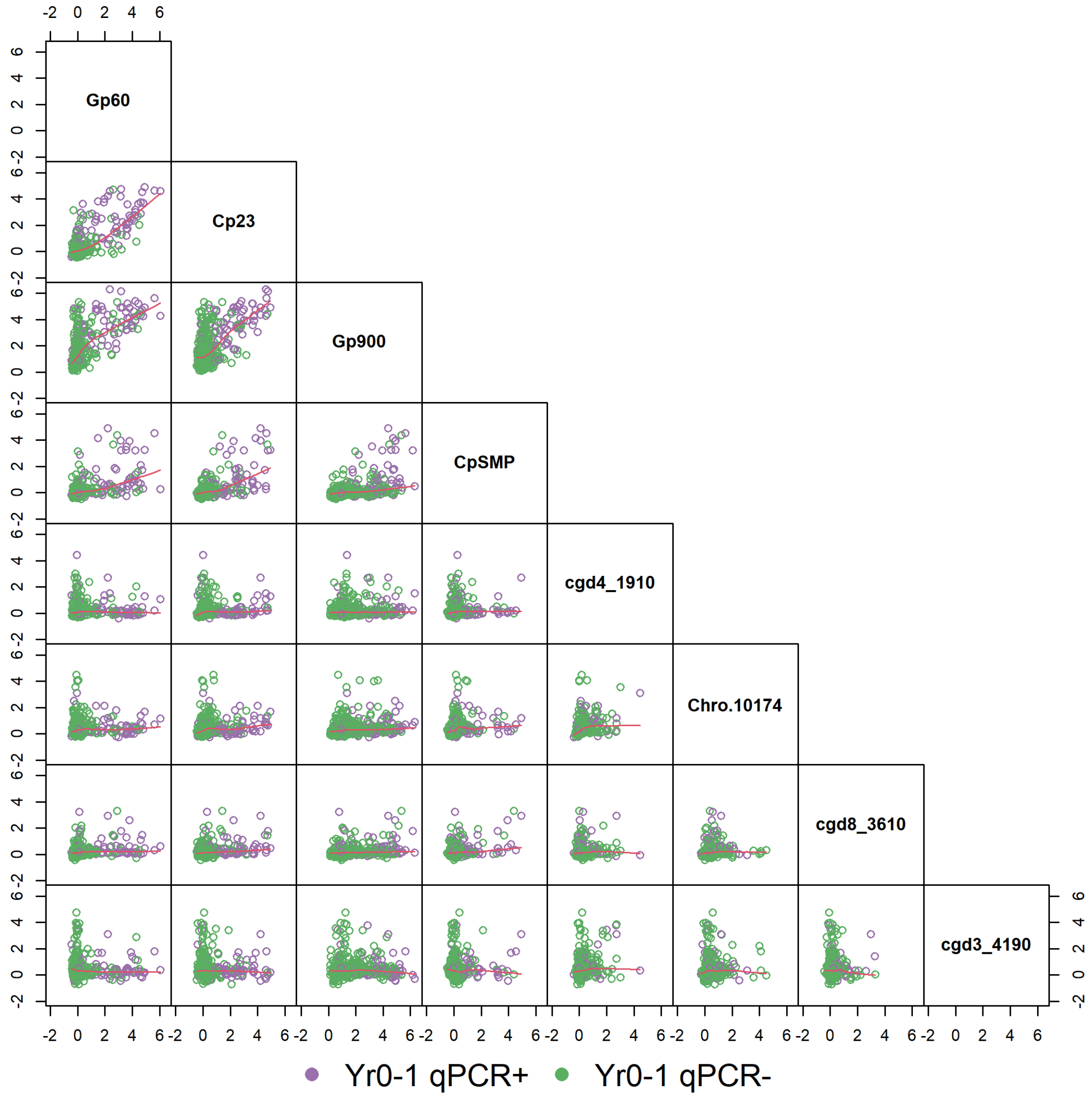
Gp60
Cp23
Gp900
CpSMP
cgd4_1910
Chro.10174
cgd8_3610
cgd3_4190
Yr0-1 qPCR+
Yr0-1 qPCR-

Supplemental Figure 11. Correlation of protective antibodies and antibodies associated with increased risk of reinfection with inflammatory and T cell activation markers for B cell help. The correlogram shows the Spearman's correlation coefficients for the comparisons between antibody levels against antigens identified in random forest analysis and HAZ scores, IL4, CRP, IL1β and sCD14 levels. Protective antigens: Gp60, Cp23, Gp900 and CpSMP; antigens associated with increased risk of subsequent infection: cd4_1910, Chro.10174, cgd8_3610 and cgd3_4190.

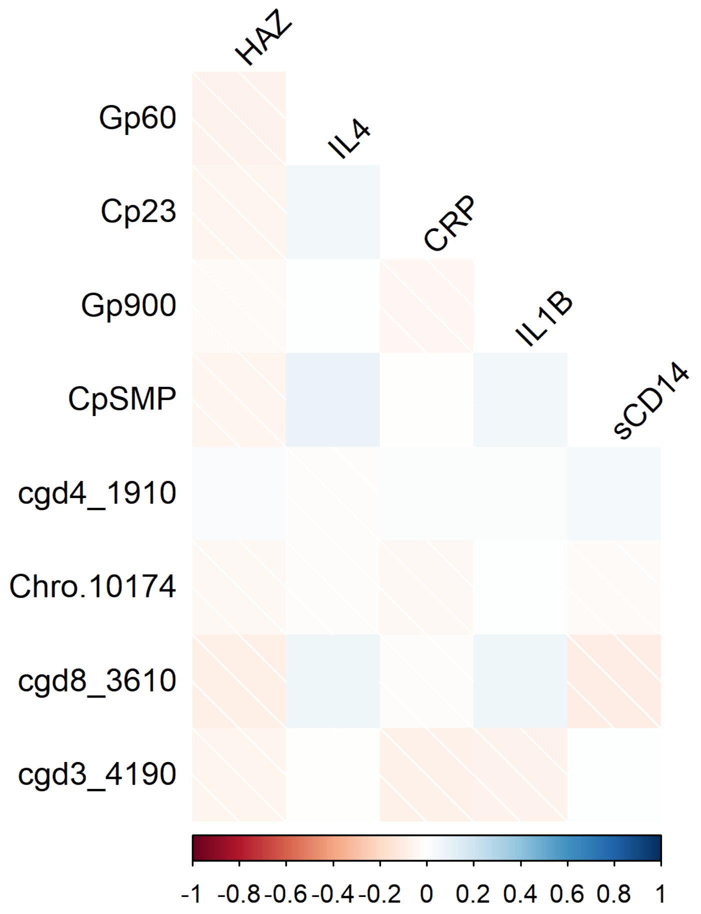

Supplemental Figure 12. Antigens associated with a delay in infection in the survival analysis but not selected by random forest analysis. The Kaplan-Meier curves show associations of seropositivity to cryptosporidial antigens with a delay in *Cryptosporidium* infection during the 1 year and 2 year follow-up periods following plasma sampling at 1 year of age. The X-axis shows days after the end of year one (when the assayed plasma sample was collected). The Y-axis shows the proportion of children who remained uninfected as defined by qPCR+ clinical or surveillance stool samples during follow-up. The tables below the graphs indicate the number of children in the seropositive (blue lines) or seronegative (red lines) category at select time points. Antigens are indicated at the top of each panel, along with the follow-up period and the antibody isotype associated with risk of infection. Hazard ratios (β), confidence intervals (CI), P-values and P-values adjusted for the false discovery rate (BH) are shown in each graph from univariate models and multivariable models adjusted for covariates (β_{adj}, CI_{adj}, P_{adj} and BH P_{adj}).

A. IgG: cgd4_1970, 1 year risk of reinfection in children with Yr0-1 qPCR+ stool samples

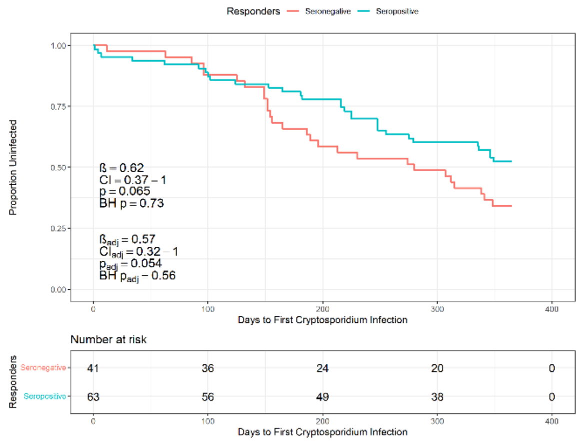

B. IgA: cgd4_1970, 1 year risk of reinfection in children with Yr0-1 qPCR+ stool samples

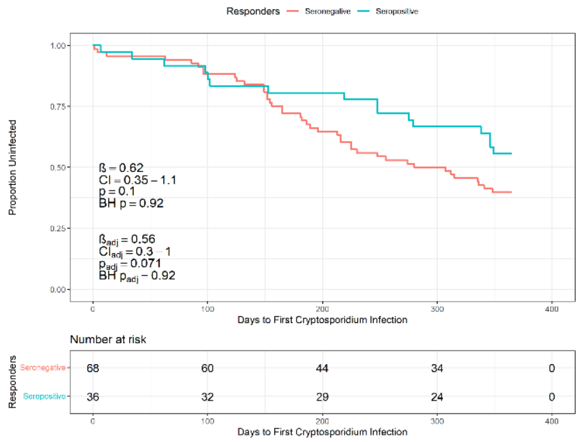

C. IgG: cgd4_1970, 2 year risk of reinfection in children with Yr0-1 qPCR+ stool samples

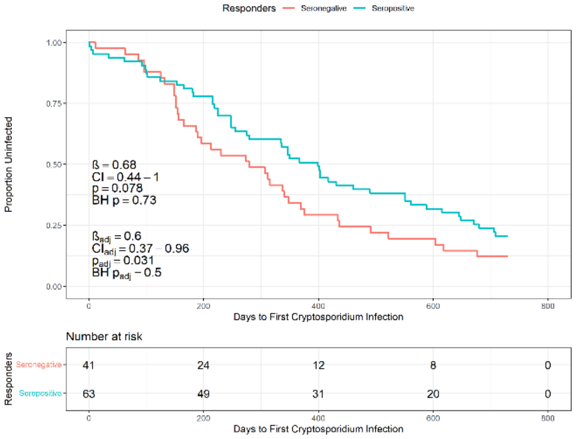

D. IgG: cgd5_1490, 2 year risk of reinfection in children with Yr0-1 qPCR+ stool samples

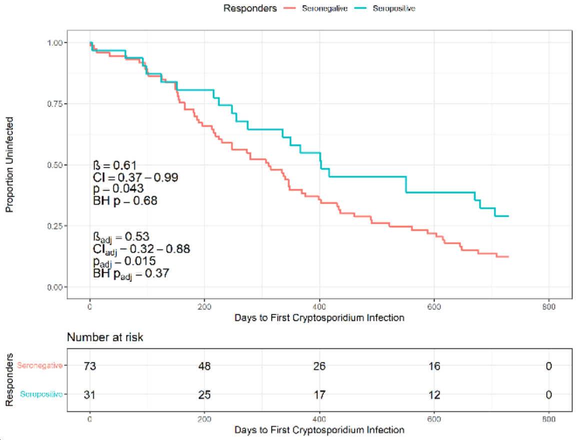

E. IgA: cgd6_4870, 2 year risk of reinfection in children with Yr0-1 qPCR+ stool samples

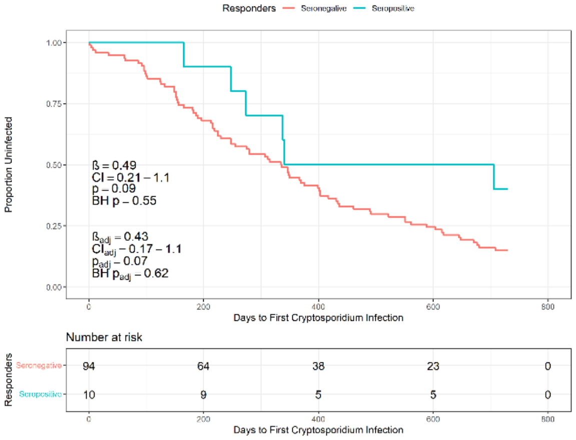

F. IgG: cgd8_3520, 2 year risk of reinfection in children with Yr0-1 qPCR+ stool samples

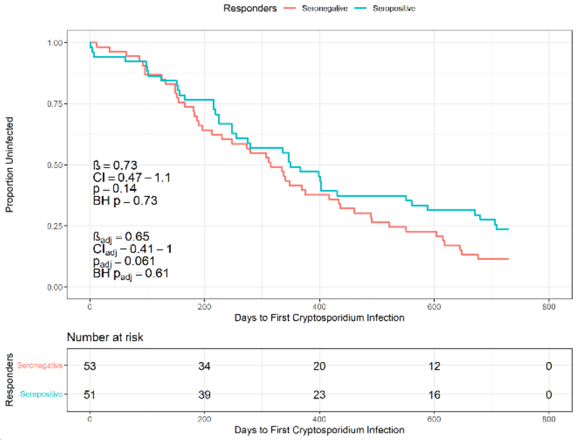

G. IgG: cgd3_1130, 1 year risk of reinfection in children with Yr0-1 qPCR+ stool samples

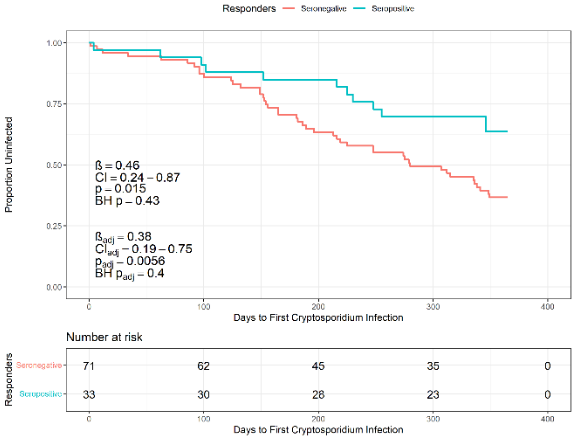

H. IgG: cgd1_650, 2 year risk of reinfection in children with Yr0-1 qPCR+ stool samples

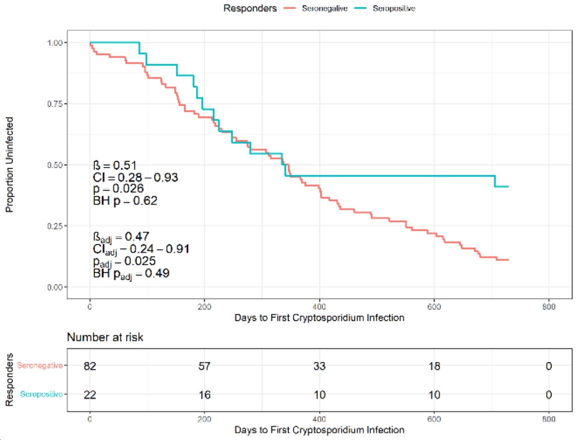

I. IgG: cgd8_660, 2 year risk of reinfection in children with Yr0-1 qPCR+ stool samples

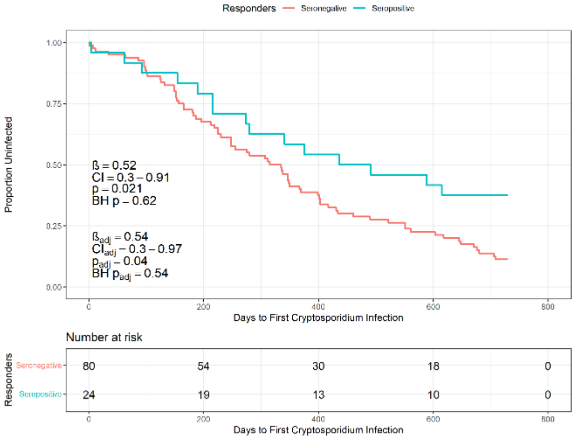

J. IgG: cgd4_1970, 2 year risk of infection in all children

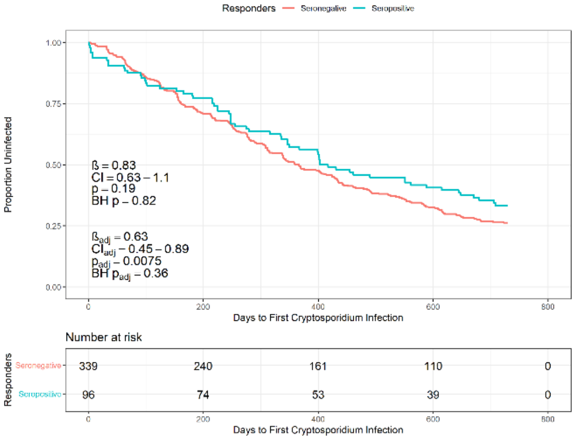

K. IgG: cgd5_1490, 1 year risk of infection in all children

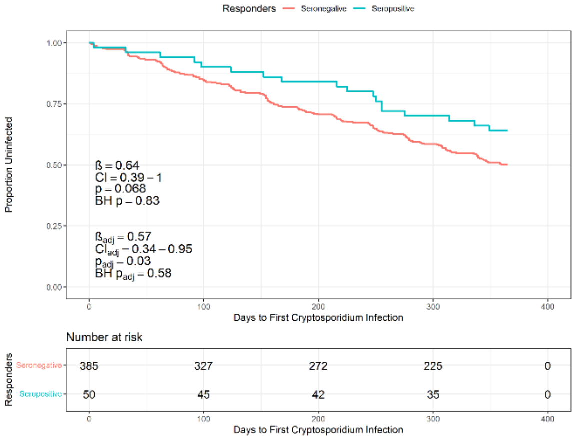

L. IgG: cgd5_1490, 2 year risk of infection in all children

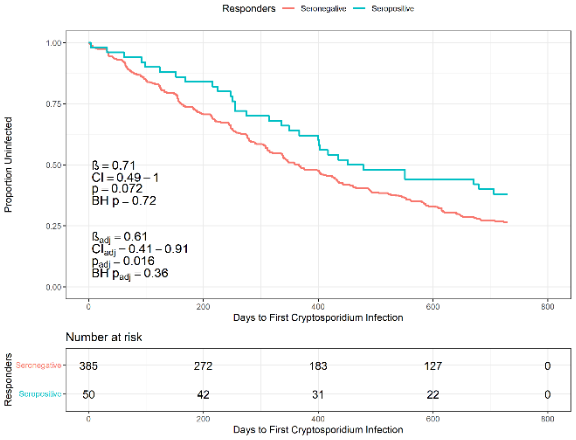

M. IgA: cgd6_4870, 2 year risk of infection in all children

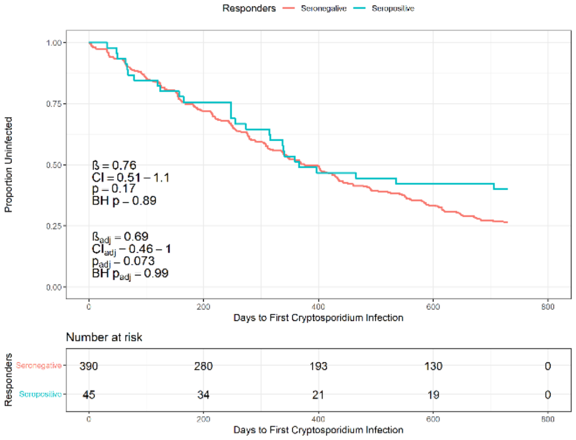

N. IgG: cgd7_2150, 1 year risk of infection in all children

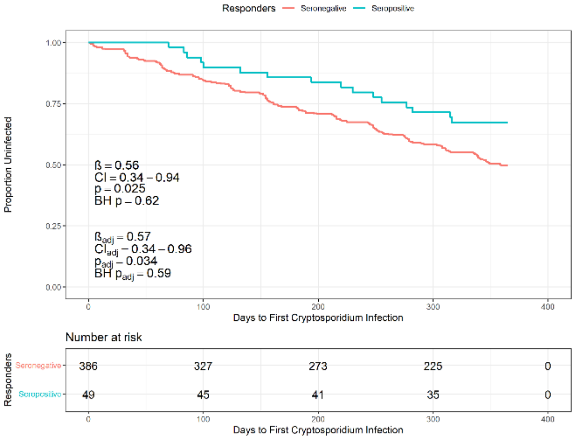

O. IgG: cgd7_2150, 2 year risk of infection in all children

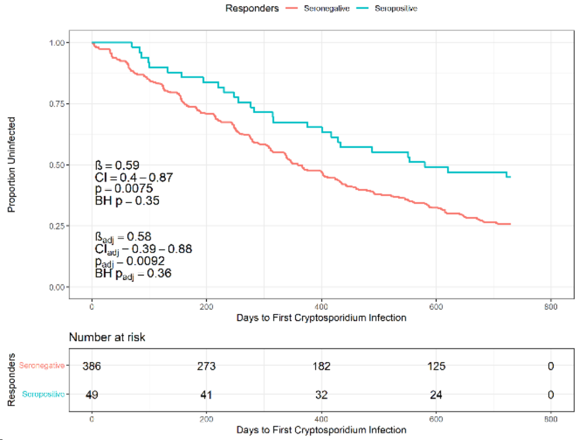

P. IgG: cgd8_3520, 2 year risk of infection in all children

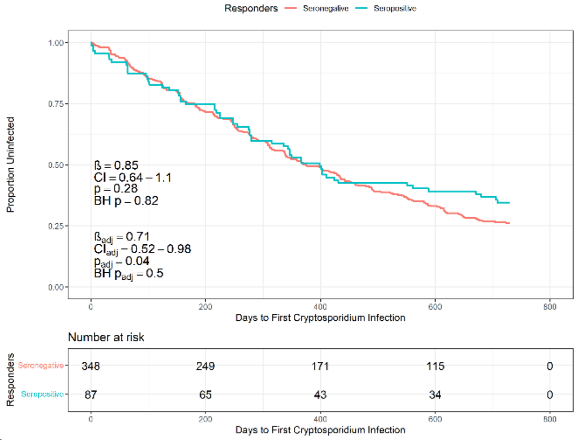

Q. IgG: cgd3_720, 2 year risk of infection in all children

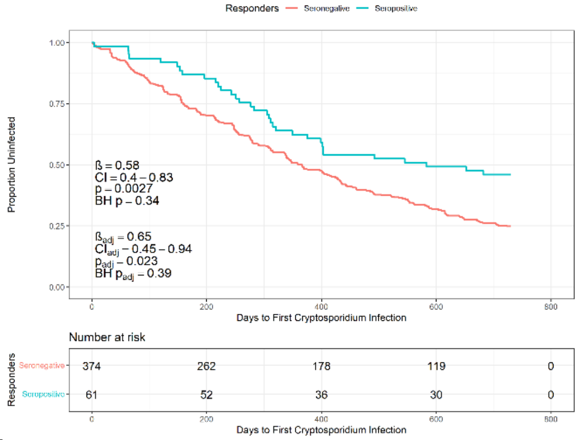

R. IgG: cgd2_1200, 2 year risk of infection in all children

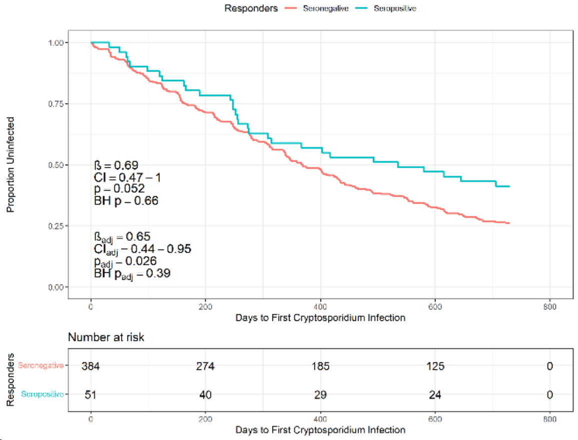

Supplement: Supplemental data [file jci-133-166814-s096.pdf]
